# Intraoperative supraglottic airway device, anesthetic, and mechanical factors associated with acute postoperative sore throat in elective tympanoplasty: a retrospective cohort study

**DOI:** 10.1186/s12871-026-04024-2

**Published:** 2026-06-16

**Authors:** Weihong Yang, Liping Wang, Yanhong Hu, Xiaomei Lou, Weixing Li, Yanzhe Huang

**Affiliations:** Department of Anesthesiology, the EYE & ENT Hospital of Fudan University, Shanghai, 200031 China

**Keywords:** Elective tympanoplasty, Laryngeal mask, Sore throat, Risk factors, Head rotation, Position change, Retrospective study

## Abstract

**Background:**

Elective tympanoplasty often uses supraglottic airway devices, such as the laryngeal mask airway (LMA), to ensure smooth emergence from anesthesia. However, LMA-associated postoperative sore throat (POST) remains prevalent. Lateral head rotation during surgery alters LMA intracuff pressure (CP), but the relationship between these pressure changes and POST remains unclear. We aimed to investigate the associations of LMA characteristics, anesthetic management, and position-induced dynamic CP variations with acute POST.

**Methods:**

This retrospective cohort study included 1,363 adult patients undergoing elective tympanoplasty with LMAs. The primary outcome was acute incident POST (visual analog scale [VAS] scores > 0) within 30 min after surgery, and the secondary outcome was POST severity (VAS scores of 0, 1–3, and ≥ 4). Multivariate regression models were used to analyze potential associations with POST. Spline, threshold, and interaction analyses were used to explore non-linear relationships and evaluate the modifying effects of anesthesia duration on CP changes.

**Results:**

The overall incidence of acute POST was 39.6% (470 mild and 70 moderate-to-severe POST). Tuoren LMA (vs. Flexible LMA), multiple insertion attempts (≥ 3), and elevated baseline supine CP had strong associations with increased risk of incident POST. Non-linear associations with POST incidence were observed for anesthesia duration, position-induced absolute CP change, and relative CP change. Furthermore, anesthesia duration and absolute CP change showed a significant interaction (*P* = 0.037), with a minor position-induced CP change (-0.52 to 5.79 cmH₂O) associated with a higher risk of acute POST in procedures lasting ≥ 1.3 h (OR: 1.146, 95% CI: 1.041–1.261), but not in shorter surgeries. Similar results were also observed for POST severity, particularly mild POST.

**Conclusion:**

This study demonstrated that specific LMA brands, repeated insertions, and elevated baseline CP were significantly associated with acute POST in elective tympanoplasty, and found that a minor position-induced CP change was associated with increased POST risk when anesthesia duration was prolonged. Future prospective trials are warranted to explore the potential complex relationships between supine CP, position-induced CP changes, and anesthesia duration and to assess the clinical need for continuously regulating LMA cuff pressure after head rotation during prolonged otologic surgeries to optimize postoperative recovery.

**Supplementary Information:**

The online version contains supplementary material available at 10.1186/s12871-026-04024-2.

## Introduction

Elective tympanoplasty is a widely performed ear surgery for restoring hearing [1]. Ideal surgical outcomes require smooth emergence from anesthesia to avoid pressure-induced displacement of the tympanic grafts [2, 3]; thus supraglottic airway devices (SADs) have become the preferred alternative to reduce airway stimulation and maintain stable hemodynamics [4, 5]. Despite less airway trauma [6–9], SAD-associated postoperative sore throat (POST) remains prevalent with a 1-h pooled incidence of 20.5%–40.2% [10]. In addition to increasing the nursing burden as a primary complaint, severe POST can induce postoperative agitation, frequent swallowing, and reflexive coughing [11, 12], potentially influencing the repair and prognosis of the tympanic cavity [2, 3].

Identifying independent and modifiable pre- and intra-operative risk factors is paramount to reduce the incidence of POST and improve patient satisfaction [10, 13]. Although several systematic reviews widely summarize the general risk profiles of developing POST (e.g., age, female sex, size and cuff pressure [CP] of SADs, duration of airway device placement, number of insertion attempts, and smoking history) [10, 13, 14], high-quality observational studies that focus on SADs such as the laryngeal mask airway (LMA) [15–17] in elective tympanoplasty and adequately control for confounders are rather lacking, whereas postoperative pain is associated with different procedures [18]. Thus, those established risk factors might not accurately assess acute POST risk in patients undergoing elective tympanoplasty, making disease-specific evidence remain needed for guiding high-risk population identification.

Additionally, lateral head and neck rotation is often necessary in elective tympanoplasty for securing optimal microscopic access [2], during which the patient’s pharyngeal airway may collapse and pharyngeal geometry would be altered [19]. This shift can significantly change the oropharyngeal leak pressure [20–22] and directly increases the intracuff pressure of the LMA [19, 23]. Previous studies reported that continuously changed cuff pressure of the endotracheal tube was associated with postoperative sore throat in laparoscopic cholecystectomy [24], but direct evidence on intracuff pressure changes caused by adjusting position (i.e., position-induced cuff pressure changes) and incident acute POST in elective tympanoplasty is limited, leading to an important knowledge gap. Furthermore, the initial cuff pressure, position-induced pressure variations, and anesthetic duration may involve complex interactions [25–27]. However, little is known about the associations of position-induced dynamic variations and prolonged anesthesia-induced cumulative effects of cuff pressure with the incidence of POST under dynamic surgical conditions in elective tympanoplasty.

In this study, we aimed to investigate the associations between supraglottic airway device (i.e., LMA) characteristics, anesthetic management, and dynamic mechanical factors and the incidence of acute POST, and to further explore potential non-linear relationships and interactions of baseline intracuff pressure, position-induced pressure changes, and anesthetic duration with acute POST, using electronic medical record data from patients undergoing elective tympanoplasty.

## Methods

### Study design and population

**Fig. 1:**
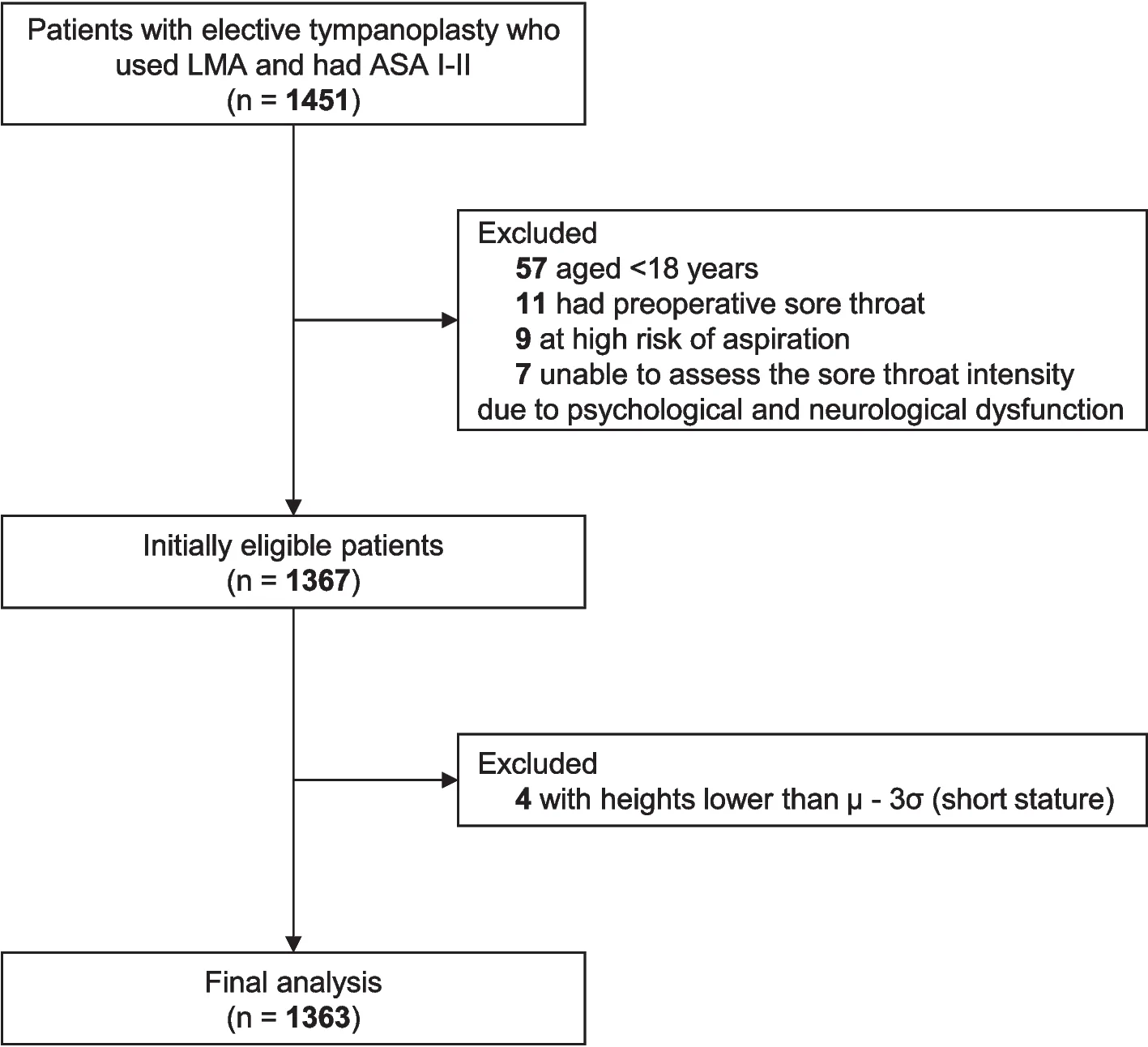
Flowchart of patients’ inclusion and exclusion in this study. ASA, American Society of Anesthesiologists physical status; LMA, laryngeal mask airway

This was a single-center, retrospective, observational cohort study conducted at the Eye & ENT hospital of Fudan University in Shanghai, which initially included 1,451 patients who underwent elective tympanoplasty, used LMAs to maintain normal ventilation, and had an American Society of Anesthesiologists physical status of I–II under general anesthesia at the otologic center between July 2019 and December 2020. Patients were excluded if they were < 18 years old, had preoperative sore throat (defined as a self-report or documented sore throat), were assessed at high risk of aspiration, or were unable to assess the intensity of sore throat owing to psychological and neurological dysfunction. Moreover, given an extremely left-tailing distribution for the patients’ height in this study, which could not be calibrated by natural logarithmic transformation, we further excluded 4 middle-aged patients with very low heights that were lower than μ—3σ (i.e., 135.6 cm), leaving 1,363 patients in the study (shown in Fig. 1).

### Anesthesia management

The whole anesthesia process followed standard procedures. Prior to anesthesia induction, anesthetists ensured no premedication was administered for each patient and then routinely monitored noninvasive blood pressure, electrocardiography, peripheral oxygen saturation, and end-tidal carbon dioxide. After preoxygenation, anesthesia was induced using 0.5 µg/kg remifentanil and 1.5–2.0 mg/kg propofol for all patients, followed by 0.6 mg/kg rocuronium or 0.1 mg/kg cis-atracurium to facilitate LMA insertion, in which lidocaine gel was used as a lubricant to ensure a smooth insertion, completed by experienced anesthetists according to the manufacturers’ instructions. The type and size of LMA were determined at the discretion of the anesthetists. Effective airway seal was achieved by recording any audible leak during manual inflation of the syringes up to 20 cmH_2_O, and the syringe was reattached to the LMA cuff to balance the plunger and cuff pressure. Adequate ventilation was determined based on normal thoracoabdominal movement and capnography end-tidal carbon dioxide tracing, and airway devices would be replaced in cases of any poor ventilation or airway seal. Next, sevoflurane- or propofol-based general anesthesia was maintained for each patient according to anesthetists’ preference, with nitrous oxide not used for any patients. Mechanical ventilation was utilized throughout the operation until the end of surgery.

### Data collection and CP measurements

Baseline information including demographics (e.g., age and sex), lifestyle (e.g., smoking status), and physical examination metrics (e.g., height and weight) were collected at admission for elective tympanoplasty by face-to-face interviews or standardized questionnaires and during physical examinations, and perioperative factors (e.g., type of LMA, size of LMA, anesthetic method, surgery ear, number of attempts, duration of surgery, and cuff pressure) were recorded during the procedures. All information was extracted from the electronic medical records by two trained nurses and randomly checked by another nurse to ensure reliability and quality of data collection. Table S1 details the definition and collection of all potential factors associated with acute POST in elective tympanoplasty.

Specifically, body mass index (BMI) was calculated as the weight in kilograms divided by the square of the height in meters. Current smoking was defined as ≥ 1 cigarette per day. The type of LMA was chosen by anesthetists according to their clinical experience and preference and recorded as four types, including the laryngeal mask airway Flexible (FLMA, LMA Flexible™, Laryngeal Mask Company Limited, Perak, Malaysia), Ambu AuraFlex LMA (Ambu-F, Ambu® AuraGain™ Laryngeal Airway, Denmark), Tuoren reinforced LMA (Tuoren-R, Tuoren Medical Company, Henan, China) [28], and Tuoren Esophagus Drainage Type I LMA (Tuoren-ED, Tuoren-R, Tuoren Medical Company, Henan, China) [28]. The size of LMA was sex-standardized and classified as size 3 and ≥ 4 (i.e., size of 4 or 5), in which size 3 was often used for women and size 4 for men. Anesthetic method was defined as the way of maintaining the status of general anesthesia and recorded as total inhalation anesthesia or intravenous anesthesia. Surgical ear was defined as the ear which required surgical operations and recorded as right, left, or both ears. Number of attempts was defined as the needed insertion number until the LMA was correctly seated with an adequate seal, and recorded as 1, 2, and ≥ 3 insertion attempts. Duration of anesthesia was defined as the time from anesthesia induction until awake extubation.

Intracuff pressure was measured for each patient using a calibrated aneroid manometer (VBM Medizintechnik, Sulz, Germany) connected to the pilot balloon valve of the LMA via a three-way stopcock to prevent inadvertent gas leakage during measurement. After LMA insertion and adequate ventilation, patients were maintained in the standard supine position (0° head elevation). Triplicate CP measurements at end-expiration (defined as the nadir of the airway pressure waveform displayed on the ventilator monitor) were taken with ≥ 30 s between successive readings, and the mean value was recorded as the supine cuff pressure. Subsequently, the patient’s position was gently adjusted by trained staff to rotate him/her head toward the side contralateral to the operative ear. After a 5-min wait, lateral cuff pressure was measured using the identical protocol applied in the supine position, with three readings at 30-s intervals, and the mean value was recorded.

To investigate the associations between position-induced CP change and cumulative pressure burden and the incidence of acute POST, four passive CP metrics were calculated, including the absolute change in CP (defined as subtracting the supine cuff pressure from the lateral cuff pressure), relative change in CP (defined as dividing the change of CP by supine cuff pressure), cumulative absolute change in CP (defined as multiplying the absolute change in CP by the duration of anesthesia), and cumulative relative change in CP (defined as multiplying the relative change in CP by the duration of anesthesia).

### Outcome assessment

The primary outcome was acute incident POST. The intensity of sore throat was assessed using the visual analog scale (VAS) scores within 30 min after surgery in the post-anesthesia care unit by trained nurses for each patient, with the points ranging from 0 to 10. Acute incident POST was defined as the postoperative 30-min VAS of higher than 0. To further explore the associations between these intraoperative risk factors and different intensities of acute POST, we defined the secondary outcome as the POST severity, which was categorized into three classes, including a postoperative 30-min VAS scores of 0 (no pain), VAS scores of 1–3 (acute mild pain), and VAS scores of 4–10 (acute moderate-to-severe pain).

### Missing data

To reduce the potential impact of high missingness on association estimation, variables with missing rates higher than 50% were firstly removed [29]. The smoking variable was specifically retained owing to its important role in POST [10, 13] despite a marginally high missingness. Table S2 provides detailed missing information. Then, missing imputation with chained equations [30, 31] was applied to impute missing data for smoking, anesthetic method, surgery ear, duration of anesthesia, and cuff pressure under the missing at random (MAR) assumption. The imputation model contained all other variables in Table S2 and the original endpoint variable (VAS), in which BMI (not imputed but specified), absolute and relative change in CP, cumulative absolute and relative change in CP entered the models through passive imputation; meanwhile, age was transformed by mean-centering and its squared term was included in imputation models. We totally generated 50 imputed datasets and used all of them in the descriptive and association analyses. All statistical results were generally pooled based on Rubin’s rule [30, 32, 33]. For any inappropriate cases like cutoffs or threshold determination, stacked imputation was used instead [34].

### Statistical analysis

Continuous variables were described as mean ± standard deviation (SD) or median [interquartile range, IQR], and categorical variables were presented as frequencies (percentages). For the primary outcome, statistical differences were evaluated by unpaired Student’s t-test or Wilcox rank-sum test and chi-square test, and for the secondary outcome, differences were compared by ANOVA analysis or Kruskal–Wallis’ test and chi-square test or Fisher’s exact test, as appropriate.

We used binary logistic regression models to estimate odds ratios (ORs) and 95% confidence intervals (CIs) of acute incident POST (primary outcome) associated with several intraoperative risk factors, including type of LMA, size of LMA, anesthesia method, number of insertion attempts, duration of anesthesia, supine cuff pressure, lateral cuff pressure, and position-induced absolute change in CP, relative change in CP, cumulative absolute change in CP, and cumulative relative change in CP. For the secondary outcome (POST severity), multinomial log-linear regression models were used.

All multivariate models were adjusted for age, age squared, female sex, smoking, and BMI, with the age-squared term used to fit potential aging-associated non-linear vulnerability [35]. Additionally, we adjusted factor-specific covariables based on previous reports and established knowledge. Specifically, for type of LMA, size of LMA, and anesthesia method, models were adjusted for age, age squared, female sex, smoking, and BMI. Number of attempts was also adjusted for type of LMA and size of LMA. For duration of anesthesia and supine cuff pressure, models were in addition adjusted for type of LMA, size of LMA, number of attempts, and anesthesia method. For lateral cuff pressure, position-induced absolute and relative change in CP, and cumulative absolute and relative change in CP, models were further adjusted for type of LMA, size of LMA, number of attempts, anesthesia method, and supine cuff pressure. To reduce nonessential collinearity between age and age squared, mean-centering was applied for age. Multicollinearity diagnostics were conducted by calculating the Variance Inflation Factor (VIF).

Moreover, each continuous variable was categorized based on the quintiles, and P for trend was calculated. We also carried out a propensity score analysis for each intraoperative factor of interest and the propensity score was calculated by the overlap weighting method. Covariate balance across the generated pseudo-populations was evaluated using the absolute standardized mean difference, with a threshold below 0.10 considered to maintain optimal equilibrium. Subsequently, the average treatment effect on the overlap population was estimated for each focused surgical metric. Subgroup analyses were performed to estimate adjusted ORs stratified by age (< 50 and ≥ 50 years), sex (male and female), BMI (< 24 and ≥ 24 kg/m^2^), and smoking status, in which P for interaction was calculated using likelihood ratio tests by comparing the full models with and without interaction terms for age > 50 years, female, BMI ≥ 24 kg/m^2^, and current smoker.

To explore potential non-linear relationships, duration of anesthesia, supine and lateral cuff pressure, absolute and relative change in CP, and cumulative absolute and relative change in CP were respectively modeled with restricted cubic splines (knots of 3–5) for the primary and secondary outcomes, in which appropriate knots were selected based on the AIC rule. For those variables with non-linear *P* < 0.1, threshold effect analyses were used to identify potential and clinically meaningful inflection points based on nonlinear likelihood ratio tests. If convergence was lacking, the age-squared term would be removed from non-linear logistic or multinomial models. Based on potential segmented thresholds, continuous variables were segmented to estimate piecewise associations. Additionally, to elucidate the effects of potential supine CP- and anesthetic duration-modifying position-induced change in CP and cumulative pressure burden on acute POST, we estimated associations between four position-induced CP metrics (including absolute and relative change in CP and cumulative absolute and relative pressure burden) and POST stratified by baseline supine cuff pressure and duration of anesthesia, and used piecewise regression models to calculate segmented ORs stratified by supine cuff pressure and duration of anesthesia.

Finally, we conducted a sensitivity analysis. Specifically, for categorical variables with missing values, we coded missingness as a separate “Unknown” category, and used it as an additional dummy-coded level in regression models, with “No”, “Inhalation”, and “Right” serving as the reference groups for smoking, anesthetic method, and surgery ear respectively. For continuous variables, a single imputation with predictive mean matching was applied.

All statistical analyses were performed using R software (version 4.5.1, R Foundation for Statistical Computing, https://cran.r-project.org/). Two-sided *P* values < 0.05 were considered statistically significant. The study adhered to the Strengthening the Reporting of Observational Studies in Epidemiology (STROBE) statement.

## Results

### Baseline characteristics

Table 1 summarizes the pooled baseline characteristics of the 1,363 included patients. The overall incidence of acute incident POST was 39.6%, comprising 470 patients with mild POST and 70 with moderate-to-severe POST. The mean age was 44.46 ± 13.34 years and 56.9% were female. Compared with the non-POST group, patients who developed acute incident POST had a higher proportion of females (64.6% *vs.* 51.9%) and lower height. The distribution of LMA types between non-POST and POST groups was different and patients in the POST group were more likely to have a selection of smaller LMAs, and have a higher level of baseline supine cuff pressure (28.81 ± 8.60 *vs.* 27.38 ± 8.79 cmH₂O) and lateral cuff pressure (28.66 ± 9.19 *vs.* 26.71 ± 9.10 cmH₂O) under general anesthesia. Similar distribution was found for POST severity (Table S3).

**Table 1:** Pooled baseline clinical characteristics of patients with or without acute POST

Variables, N (%)	Overall (*N* = 1363)	Non-POST (*N* = 823)	POST (*N* = 540)
Age, years	44.46 ± 13.34	44.01 ± 13.52	45.14 ± 13.03
Female	776 (56.9)	427 (51.9)	349 (64.6)
Smoking	157 (11.5)	106 (12.9)	50 (9.3)
Weight, kg	62.94 ± 11.64	63.17 ± 11.45	62.58 ± 11.93
Height, cm	164.74 ± 7.94	165.37 ± 7.91	163.78 ± 7.88
BMI, kg/m^2^	23.09 ± 3.25	23.00 ± 3.20	23.21 ± 3.32
Type of LMA			
FLMA	602 (44.2)	432 (52.5)	170 (31.5)
Ambu-F	35 (2.6)	28 (3.4)	7 (1.3)
Tuoren-R	198 (14.5)	119 (14.5)	79 (14.6)
Tuoren-ED	528 (38.7)	244 (29.6)	284 (52.6)
Size of LMA			
3	750 (55.0)	415 (50.4)	335 (62.0)
4 or 5	613 (45.0)	408 (49.6)	205 (38.0)
Anesthetic method			
Inhalation	226 (16.6)	123 (15.0)	102 (18.9)
Intravenous	1137 (83.4)	700 (85.0)	438 (81.1)
Surgery ear			
Right	668 (49.0)	390 (47.4)	279 (51.6)
Left	672 (49.3)	419 (51.0)	252 (46.7)
Both	23 (1.7)	14 (1.7)	9 (1.7)
Number of attempts			
1	1280 (93.9)	780 (94.8)	500 (92.6)
2	65 (4.8)	36 (4.4)	29 (5.4)
3	18 (1.3)	7 (0.9)	11 (2.0)
Duration of anesthesia, h	1.29 [0.97, 1.78]	1.28 [0.95, 1.82]	1.30 [0.97, 1.72]
Intracuff pressure, cmH_2_O			
Supine	27.95 ± 8.74	27.38 ± 8.79	28.81 ± 8.60
Lateral	27.48 ± 9.18	26.71 ± 9.10	28.66 ± 9.19
Absolute Δ	0.00 [−4.00, 4.00]	0.00 [−4.00, 4.00]	0.00 [−4.00, 4.00]
Relative Δ	0.00 [−16.67, 18.18]	0.00 [−17.80, 18.02]	0.00 [−14.55, 18.18]
Cumulative absolute Δ	0.00 [−6.30, 5.15]	0.00 [−6.47, 4.96]	0.00 [−6.00, 5.60]
Cumulative relative Δ	0.00 [−21.10, 21.81]	0.00 [−22.67, 20.45]	0.00 [−19.51, 23.64]

For continuous variables with normality, we calculated the pooled mean (standard deviation) based on Rubin’s rules, and median [interquartile range, IQR] and frequencies (percentages) were pooled as the average values across 50 imputed datasets

Absolute and relative Δ refer to absolute and relative change in CP, whereas cumulative absolute and relative Δ refer to cumulative absolute and relative change in CP, respectively

*Ambu-F* Ambu AuraFlex; BMI, body mass index, *FLMA* Laryngeal mask airway Flexible, *LMA* Laryngeal mask airway, *POST* Postoperative sore throat, *Tuoren-ED* Tuoren Esophagus Drainage Type I, *Tuoren-R* Tuoren Reinforced Type

### Relationships between intraoperative factors and acute POST

Univariate regression results of potential intraoperative risk factors are provided in Table S4. Multicollinearity diagnostics rejected problematic multicollinearity among these intraoperative factors (Table S5). In the multivariate models, the type of LMA had a strong association with POST incidence and severity (Table 2). Compared to FLMA, the Tuoren-R (OR = 1.673, 95% CI: 1.190–2.351) and Tuoren-ED (OR = 3.418, 2.602–4.491) were significantly associated with an increased risk of acute incident POST, with higher risk observed for moderate-to-severe POST. Multiple insertion attempts (≥ 3) were associated with higher POST incidence (OR = 3.206, 1.149–8.944) and moderate-to-severe POST (OR = 7.574, 1.475–38.900), but not with mild POST (*P* = 0.065). Regarding mechanical pressure metrics, higher supine cuff pressure was associated with elevated risk of POST incidence and the adjusted OR was 1.019 (95% CI: 1.005, 1.032). Four passive metrics were not significantly associated with POST incidence and severity.

**Table 2:** Adjusted odds ratios for associations between intraoperative factors and POST incidence and severity based on 50 imputations

Factors	POST incidence		POST severity			
			**Mild**		**Moderate-to-severe**	
	**OR (95% CI)**	***P* value**	**OR (95% CI)**	***P* value**	**OR (95% CI)**	***P* value**
Type of LMA						
FLMA	1.000 (Ref)		1.000 (Ref)		1.000 (Ref)	
Ambu-F	0.503 (0.214, 1.180)	0.114	0.539 (0.229, 1.266)	0.156	N.A.	N.A.
Tuoren-R	1.673 (1.190, 2.351)	0.003	1.557 (1.097, 2.210)	0.013	4.060 (1.523, 10.828)	0.005
Tuoren-ED	3.418 (2.602, 4.491)	< 0.001	2.842 (2.144, 3.767)	< 0.001	16.867 (7.656, 37.157)	< 0.001
Size of LMA						
3	1.000 (Ref)		1.000 (Ref)		1.000 (Ref)	
4 or 5	0.941 (0.568, 1.558)	0.812	1.096 (0.651, 1.847)	0.730	0.277 (0.075, 1.022)	0.054
Anesthetic method						
Inhalation	1.000 (Ref)		1.000 (Ref)		1.000 (Ref)	
Intravenous	0.776 (0.508, 1.184)	0.237	0.850 (0.571, 1.265)	0.421	0.470 (0.172, 1.284)	0.139
Number of attempts						
1	1.000 (Ref)		1.000 (Ref)		1.000 (Ref)	
2	1.509 (0.891, 2.557)	0.126	1.667 (0.988, 2.815)	0.056	N.A.	N.A.
3	3.206 (1.149, 8.944)	0.026	2.759 (0.939, 8.110)	0.065	7.574 (1.475, 38.900)	0.015
Duration of surgery	1.020 (0.870, 1.195)	0.811	1.017 (0.865, 1.197)	0.835	1.068 (0.737, 1.548)	0.726
Intracuff pressure						
Supine	1.019 (1.005, 1.032)	0.006	1.016 (1.002, 1.030)	0.022	1.036 (1.005, 1.069)	0.023
Lateral	1.012 (0.994, 1.030)	0.193	1.012 (0.994, 1.030)	0.210	1.008 (0.967, 1.052)	0.694
Absolute Δ	1.012 (0.994, 1.030)	0.193	1.012 (0.994, 1.030)	0.210	1.008 (0.967, 1.052)	0.694
Relative Δ	1.000 (0.997, 1.004)	0.808	1.000 (0.997, 1.004)	0.892	1.001 (0.992, 1.011)	0.780
Cumulative absolute Δ	1.012 (1.001, 1.024)	0.032	1.011 (1.000, 1.023)	0.053	1.019 (0.992, 1.047)	0.175
Cumulative relative Δ	1.001 (0.999, 1.003)	0.271	1.001 (0.999, 1.003)	0.422	1.003 (0.999, 1.007)	0.141

All logistic and multinomial models were adjusted for age, age squared, female sex, smoking, and BMI. Additionally, each model was further adjusted for type of LMA, size of LMA, anesthetic method, and number of attempts for duration of anesthesia and supine cuff pressure, and was adjusted for type of LMA, size of LMA, anesthetic method, number of attempts, and supine cuff pressure for lateral cuff pressure, position-induced absolute and relative change in CP, and cumulative absolute and relative change in CP. All results were pooled based on Rubin’s rules

Absolute and relative Δ refer to absolute and relative change in CP, whereas cumulative absolute and relative Δ refer to cumulative absolute and relative change in CP, respectively

*Ambu-F* Ambu AuraFlex, *FLMA* LMA Flexible, *LMA* Laryngeal mask airway, *N.A.* Not available, *POST* Postoperative sore throat, *Tuoren-ED* Tuoren Esophagus Drainage Type I, *Tuoren-R* Tuoren Reinforced Type

When continuous factors were categorized into quintiles, we found an explicit linear trend for categorized supine cuff pressure, with the highest OR of 1.837 (1.244, 2.712) in the fourth group (30 to 37 cmH₂O). Similar results were also observed for mild and moderate-to-severe POST. Besides, associations between categorized duration of anesthesia, lateral cuff pressure, and position-induced change and cumulative change in CP and mild and moderate-to-severe POST suggested potential and complex non-linear relationships (Table S6). To minimize potential confounding biases, we conducted a propensity score analysis via overlap weighting and confirmed excellent covariate balance across groups (Figure S1). The adjusted ORs in the overlap-weighted populations remained consistent with unweighted results, as presented in Table S7. Moreover, similar relationships were noted in subgroup analyses stratified by age, sex, BMI, and smoking status (Table S8, Table S9, Table S10 and Table S11).

### Exploratory analyses

**Fig. 2:**
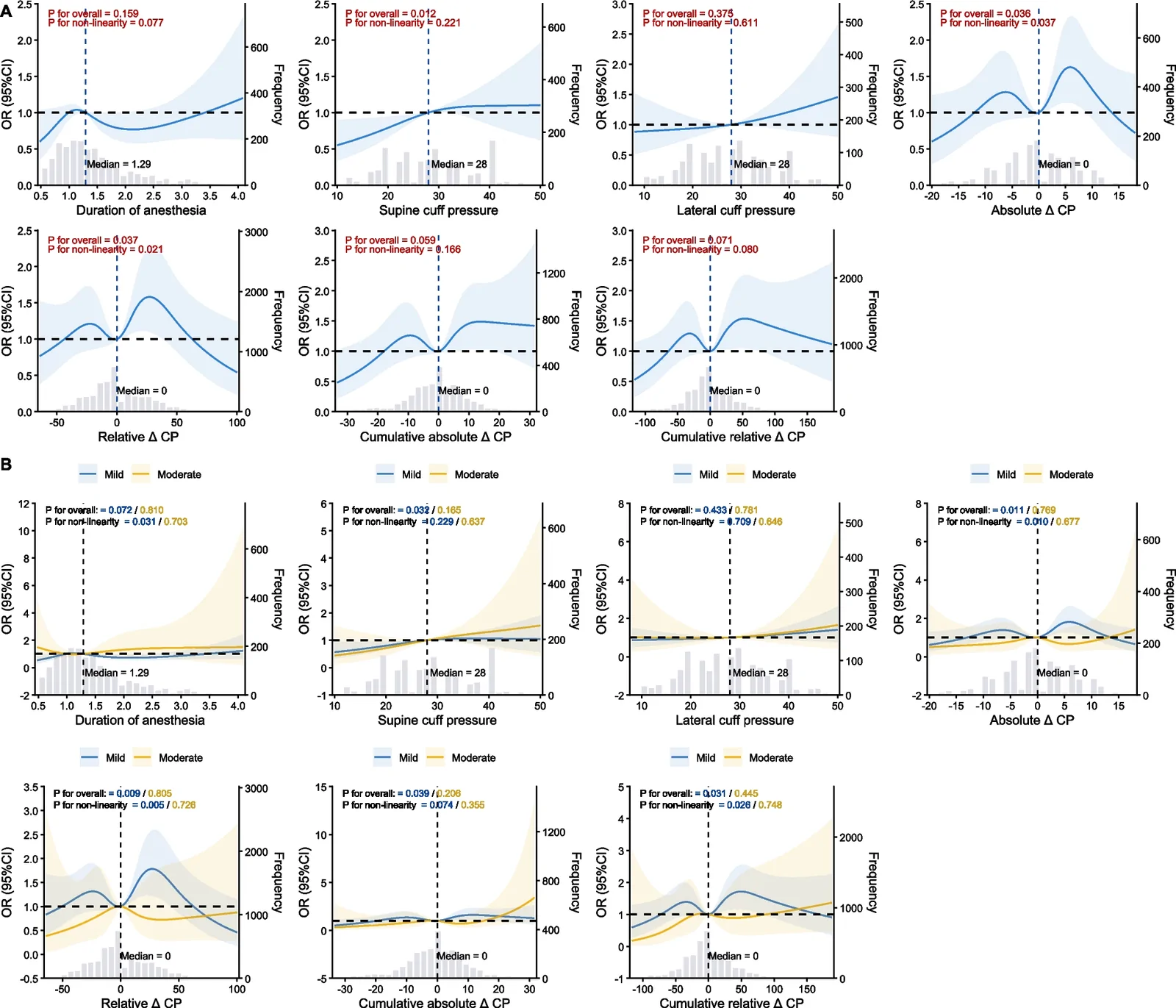
Non-linear associations of surgery duration and intracuff pressure with POST incidence and severity based on 50 imputations. **A** POST incidence. **B** POST severity. CP, cuff pressure; POST, postoperative sore throat. Restricted cubic splines (3–5 knots) of duration of surgery and intracuff pressure were separately constructed in the logistic and multinomial models for each outcome, in which all models were adjusted for age, age squared, female sex, smoking, BMI, type of LMA, size of LMA, anesthetic method, and number of attempts. Additionally, for lateral cuff pressure, absolute and relative change in CP, and cumulative absolute and relative change in CP, models were also adjusted for supine cuff pressure. The change of CP was calculated by subtracting the supine cuff pressure from the lateral cuff pressure and the change rate of CP was calculated by dividing the change of CP by supine cuff pressure

To explore potential non-linear trends with acute POST risk, we performed spline analyses and found significant non-linear associations of duration of anesthesia (“reverse S-shape”; *P* for non-linearity = 0.077 and 0.031 respectively), absolute change in CP (“M-shaped”; *P* for non-linearity = 0.037 and 0.010 respectively), relative change in CP (“M-shape”; *P* for non-linearity = 0.021 and 0.005 respectively), and cumulative relative change in CP (“M-shape”; *P* for non-linearity = 0.080 and 0.026 respectively) with acute incident POST and mild POST, as shown in Fig. 2. The non-monotonic trajectories were consistent across anesthesia states and conditions involving different LMA types, anesthetic methods, and number of attempts (Figure S2 and Figure S3). In threshold analyses, we found several potential inflection points for duration of anesthesia, absolute change in CP, relative change in CP, and cumulative relative changes (Table 3). When the duration of anesthesia was < 1.13 h (< 68 min), between 1.13 and 2.13 h (68–128 min), and ≥ 2.13 h (≥ 128 min), the OR per 1-h anesthetic time of acute incident POST was 2.309 (1.035, 5.156), 0.620 (0.408, 0.942), and 1.389 (0.938, 2.058) respectively. For absolute change in CP, we noted a small-to-moderate cuff pressure change from supine to lateral position (−0.52 to 5.79 cmH₂O) was also associated with acute incident POST (OR = 1.096, 1.024–1.174) and mild POST (OR = 1.120, 1.044–1.202). Similar results were observed for relative CP changes and cumulative relative changes.

**Table 3:** Threshold analysis of the associations of duration of surgery and position-induced change and cumulative change in CP with POST incidence and severity based on 50 imputations

Factors	POST incidence			POST severity		
	**Segment**	**OR (95% CI) per one-unit increase**	***P* value**	**Mild**		
				**Segment**	**OR (95% CI) per one-unit increase**	***P* value**
Duration of anesthesia						
	< 1.13	2.309 (1.035, 5.156)	0.041	< 1.13	2.696 (1.171, 6.208)	0.020
	1.13 to 2.13	0.620 (0.408, 0.942)	0.025	1.13 to 2.15	0.570 (0.372, 0.874)	0.010
	≥ 2.13	1.389 (0.938, 2.058)	0.101	≥ 2.15	1.479 (0.982, 2.227)	0.061
Absolute change in CP						
	< −6.25	1.055 (0.992, 1.121)	0.087	< −6.44	1.062 (0.996, 1.133)	0.065
	−6.25 to −0.52	0.945 (0.875, 1.020)	0.147	−6.44 to −0.52	0.929 (0.860, 1.003)	0.060
	−0.52 to 5.79	1.096 (1.024, 1.174)	0.008	−0.52 to 5.79	1.120 (1.044, 1.202)	0.002
	≥ 5.79	0.933 (0.869, 1.002)	0.057	≥ 5.79	0.918 (0.852, 0.990)	0.027
Relative change in CP						
	< −22.14	1.010 (0.991, 1.030)	0.291	< −23.79	1.013 (0.992, 1.034)	0.220
	−22.14 to −1.48	0.988 (0.968, 1.009)	0.253	−23.79 to −1.48	0.983 (0.964, 1.003)	0.094
	−1.48 to 27.45	1.020 (1.005, 1.035)	0.009	−1.48 to 27.45	1.026 (1.010, 1.041)	0.001
	≥ 27.45	0.984 (0.972, 0.997)	0.013	≥ 27.45	0.980 (0.967, 0.994)	0.005
Cumulative relative change in CP						
	< −31.00	1.010 (1.001, 1.020)	0.040	< −34.05	1.011 (1.000, 1.022)	0.042
	−31.00 to 0.41	0.991 (0.978, 1.003)	0.147	−34.05 to −0.41	0.988 (0.976, 1.001)	0.062
	0.41 to 53.11	1.008 (1.000, 1.017)	0.060	−0.41 to 50.05	1.012 (1.003, 1.021)	0.012
	≥ 53.11	0.998 (0.992, 1.004)	0.512	≥ 50.05	0.995 (0.988, 1.003)	0.210

All logistic and multinomial models were adjusted for age, age squared, female sex, smoking, and BMI. Additionally, each model was further adjusted for type of LMA, size of LMA, anesthetic method, and number of attempts for duration of anesthesia and supine cuff pressure, and was adjusted for type of LMA, size of LMA, anesthetic method, number of attempts, and supine cuff pressure for lateral cuff pressure, position-induced absolute and relative change in CP, and cumulative absolute and relative change in CP. The inflection points for segmentation were determined by stacking all completed datasets. All results were pooled based on Rubin’s rules

*CP* Cuff pressure, *POST* Postoperative sore throat

Additionally, when stratified by baseline supine CP, we found that a position-induced absolute CP change was associated with acute incident and mild POST in the lower baseline CP group (< 28 cmH₂O) despite no statistical interaction (*P* for interaction = 0.112 and 0.111 respectively; Table S12 and Table S13). Strikingly, a significant interaction was detected between the duration of anesthesia and position-induced CP changes for POST incidence (*P* for interaction = 0.037 for absolute change; Table S14). In procedures lasting ≥ 1.3 h (~ 78 min), an absolute CP change of −0.52 to 5.79 cmH₂O significantly elevated the risk of POST (OR: 1.146, 95% CI: 1.041–1.261, *P* = 0.006); however, this identical pressure change did not yield a significant risk of POST in shorter surgeries (< 1.3 h, OR: 1.049, 0.948–1.159, *P* = 0.354). Similar results were noted for mild POST, as provided in Table S15.

### Sensitivity analysis

Table S16 and Table S17 present the descriptive and multivariate regression results of several intraoperative factors for POST incidence and POST severity under the “Unknown”-category strategy. Generally, under this missing data processing, we found similarly consistent results in terms of baseline description and associations, to some extent suggesting the robustness of our findings.

## Discussion

In this study, we retrospectively evaluated the associations between supraglottic airway device characteristics, anesthetic management, and dynamic mechanical factors and acute POST in patients with elective tympanoplasty. We found the overall acute POST incidence was 39.6%. Type of LMA, multiple insertion attempts, and elevated supine cuff pressure were independently associated with an increased risk of developing acute POST. Moreover, relationships of position-induced CP change and anesthesia duration with acute POST may be non-linear, with several significant inflection points identified, and interactions were observed between a minor position-induced CP change and prolonged anesthesia duration. These findings highlighted the need to focus on airway device, anesthetic management, and dynamic mechanical factors to prevent acute POST in elective tympanoplasty.

Though perceived as a minor and self-limiting complication associated with postoperative discomfort and reduced satisfaction [36], POST is generally not an ideal otologic surgical outcome for elective tympanoplasty [2, 3, 11]. However, current observational studies focusing on POST are often limited by inadequate control for confounders (e.g., cuff pressure and supraglottic airway devices) [10] or Table 2 fallacy [37], and few studies concentrated on patients with elective tympanoplasty, leading to an important specific knowledge gap. Our study revealed a relatively complete perioperative risk profile of POST following general anesthesia in elective tympanoplasty, similar to large-scale systematic reviews [10, 13, 14]. For example, we found Tuoren LMAs (particularly the Esophagus Drainage Type) had a substantially higher POST risk compared to the Flexible LMA and Ambu AuraFlex LMA, even when other potential factors remained relatively low-risk levels. Previous studies reported this similar finding [22, 38, 39] and a systematic review further summarized the association between SAD brands and adverse outcomes (including POST), potentially suggesting some brand-related factors (e.g., cuff stiffness, geometry, or material) may also contribute to higher risk of acute POST [40, 41].

A distinctive feature of elective tympanoplasty is the requirement for lateral head and neck rotation to secure microscopic surgical access [2]. Previous studies suggested that such a supine-to-prone position change, which was likely to distort the oropharyngeal geometry, could not only significantly reduce oropharyngeal leak pressures [20–22] but also displace the LMA cuff [23]. Park et al. also reported that a 45-degree head rotation would cause a significant increase in cuff pressure [19] and thus potentially increase the risk of developing POST [42]. Our study demonstrated the association and more importantly, found a complex non-linear pattern where even a slight position-induced CP change (−0.52 to 5.79 cmH₂O) may be associated with acute POST, especially when supine cuff pressure was below 28 cmH₂O. Additionally, we noted that when anesthesia duration was within a certain time interval (i.e., < 68 min or ≥ 128 min), the risk of acute POST was lower than that in patients with shorter and longer anesthesia. Partly, these interesting, time threshold-based exploratory findings might suggest the importance of maintaining stable anesthetic states and appropriate anesthetic time in anesthesia management, although its fundamental reasons and mechanisms still await further investigation considering the influence of potential residual confounding, differences in case complexity, emergence characteristics, or sparse data within strata.

Moreover, we observed a significant dynamic CP change-anesthesia duration interaction, with a minor supine-to-lateral CP change (−0.52 to 5.79 cmH_2_O) significantly associated with acute POST when the procedure lasted longer than 1.3 h but not significant in shorter surgeries. After patient repositioning, prolonged exposure to even a minor CP change may also contribute to an elevated risk of developing acute incident POST. Additionally, previous studies reported that when local mechanical pressure gradually increased to exceed capillary perfusion pressure [43, 44], mucosal perfusion progressively declined, along with possible ischemic complications, potentially supporting our findings. However, given the inherent limitations of retrospective observational studies, our interaction findings should be understood and interpreted cautiously. Nevertheless, further investigation into whether continuous intraoperative cuff pressure monitoring and adjustment reduce the risk of POST in prolonged otologic procedures remains necessary [24, 45].

### Limitations

This study has several limitations. First, some perioperative variables, including smoking status, anesthetic method, and surgical ear, had relatively high missingness. Though 50 imputations using MICE were performed to try to address this problem, we acknowledge that the limited sample size (n = 1,363) and collected information might be inadequate for constructing completely correct multiple imputation models under the MAR assumption. Notably, consistent results between the 50-imputation MICE and “Unknown”-category coding strategies implicitly suggested the robustness of our findings. Second, detailed information regarding LMA operators or anesthesiologists was not recorded and was unavailable, which may influence LMA use and anesthetic management. However, in this study, all patients were managed in a single otologic surgical center under institutional anesthesia practice, reducing the variability in airway management. Third, information on analgesic administration immediately before VAS assessment was limited. Because POST was assessed early in the post-anesthesia care unit (within 30 min), residual analgesic effects may influence the absolute VAS scores. Fourth, intracuff pressure, especially lateral cuff pressure, usually changes dynamically throughout the operation due to mechanical and other factors. Mean cuff pressure based on three records might not reflect the comprehensive status of intracuff pressure. Further investigation focusing on dynamic CP is needed. Finally, this is a retrospective cohort study without later follow-up for POST or other satisfaction outcomes, which influences the establishment of definitive causal relationships due to potential residual confounding and the generalization of our findings to later related outcomes. Future prospective investigations in large-scale cohort studies or clinical trials with long-term follow-up are warranted.

## Conclusions

We systematically found several critical intraoperative risk factors independently associated with acute POST in patients undergoing elective tympanoplasty, such as specific LMA brands, multiple insertion attempts, and elevated baseline intracuff pressure, and further found complex non-linear relationships and interactions between position-induced CP change and surgical duration. Future prospective studies are warranted to evaluate whether continuous monitoring and active adjustment of intracuff pressure after head rotation are useful to optimize postoperative recovery in routine anesthetic management for elective tympanoplasty.

## Supplementary Information

Supplementary Material 1.

## Data Availability

The datasets used and/or analyzed during the current study are available from the corresponding author on reasonable request.

## Abbreviations

Ambu-F: Ambu AuraFlex
BMI: Body Mass Index
CP: Cuff Pressure
FLMA: Laryngeal Mask Airway Flexible
LMA: Laryngeal Mask Airway
POST: Postoperative Sore Throat
RCS: Restricted Cubic Spline
SAD: Supraglottic Airway Device
Tuoren-ED: Tuoren Esophagus Drainage Type I
Tuoren-R: Tuoren Reinforced Type
VAS: Visual Analog Scale
