# Supplementary material for: Intraoperative supraglottic airway device, anesthetic, and mechanical factors associated with acute postoperative sore throat in elective tympanoplasty: a retrospective cohort study
Source: BMC Anesthesiol. 2026 Jun 16;26:522. doi: 10.1186/s12871-026-04024-2 (PMC13505008; doi:10.1186/s12871-026-04024-2)
Supplement: Supplementary file 1 [file 12871_2026_4024_MOESM1_ESM.pdf]

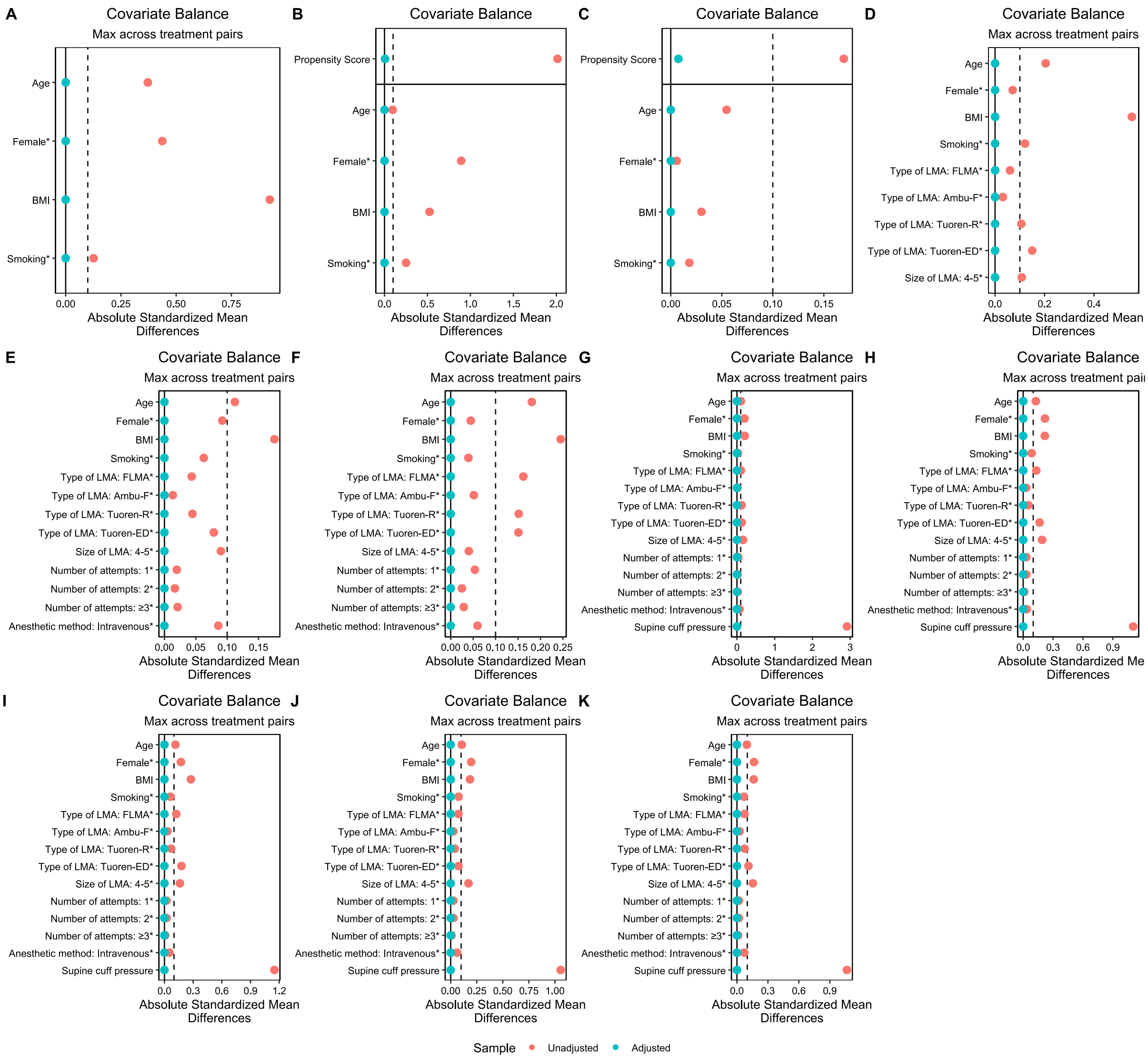

Figure S1. Covariate balance plots of intraoperative factors before and after overlap weighting

(A) Type of LMA. (B) Size of LMA. (C) Anesthetic method. (D) Number of attempts. (E) Duration of anesthesia. (F) Supine cuff pressure. (G) Lateral cuff pressure. (H) Absolute change in CP. (I) Relative change in CP. (J) Cumulative absolute change in CP. (K) Cumulative relative change in CP. Continuous variables were categorized based on their quintiles. The covariate balance plots were very similar across 50 imputed data sets, so we used the plots from the first completed dataset. Ambu-F, Ambu AuraFlex; BMI, body mass index; CP, cuff pressure; FLMA, laryngeal mask airway Flexible; LMA, laryngeal mask airway; POST, postoperative sore throat; Tuoren-ED, Tuoren Esophagus Drainage Type I; Tuoren-R, Tuoren Reinforced Type.

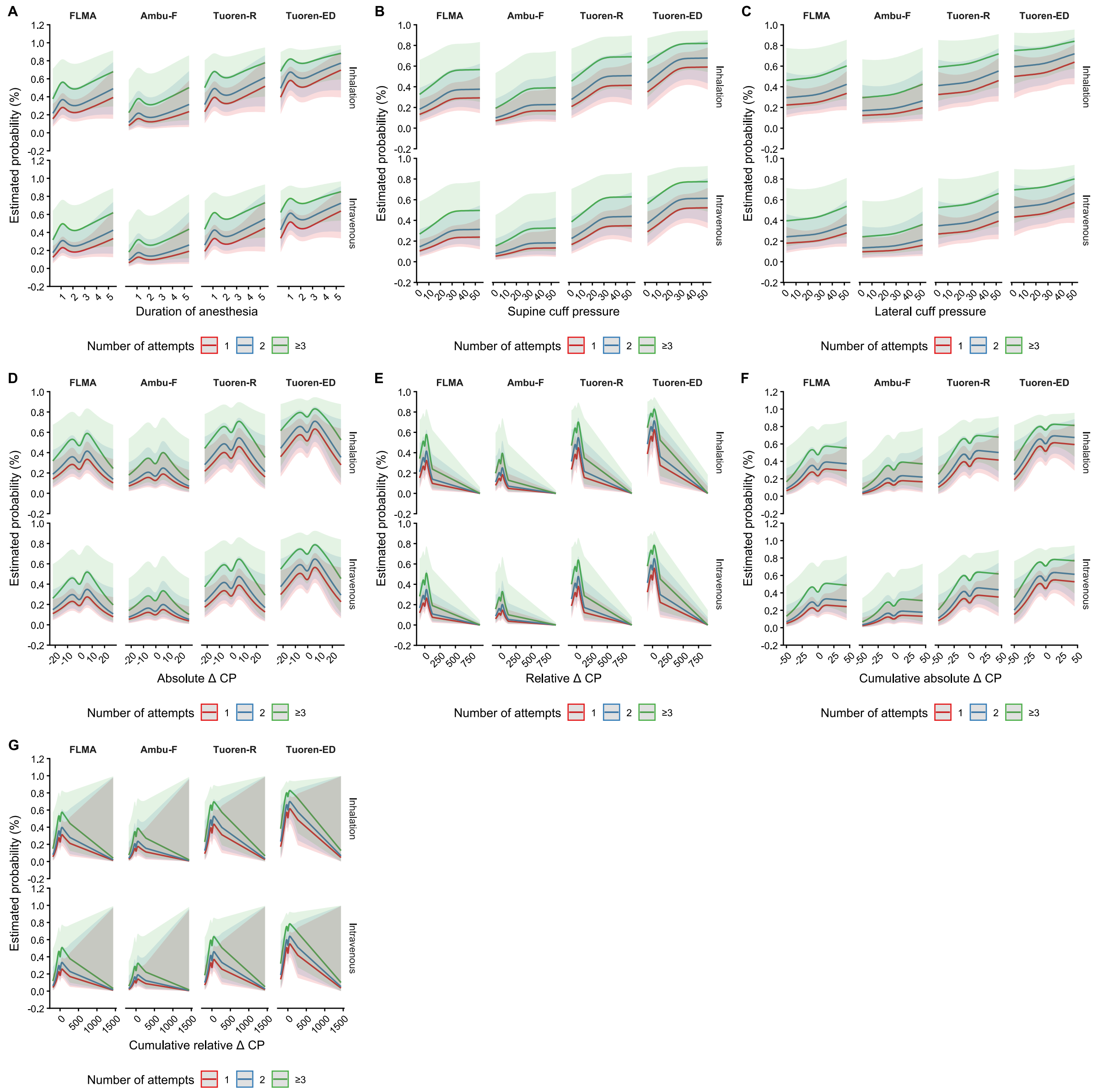

Figure S2. Adjusted predicted probabilities of acute incident POST across duration of anesthesia and intracuff pressure metrics based on 50 imputations

(A) Duration of anesthesia. (B) Supine cuff pressure. (C) Lateral cuff pressure. (D) Absolute change in CP. (E) Relative change in CP. (F) Cumulative absolute change in CP. (G) Cumulative relative change in CP.

Adjusted probabilities were estimated using the logistic models, in which duration of anesthesia, supine and lateral cuff pressure, absolute and relative change in CP, and cumulative absolute and relative change in CP were modeled with restricted cubic splines.

Ambu-F, Ambu AuraFlex; CP, cuff pressure; FLMA, laryngeal mask airway Flexible; LMA, laryngeal mask airway; POST, postoperative sore throat; Tuoren-ED, Tuoren Esophagus Drainage Type I; Tuoren-R, Tuoren Reinforced Type.

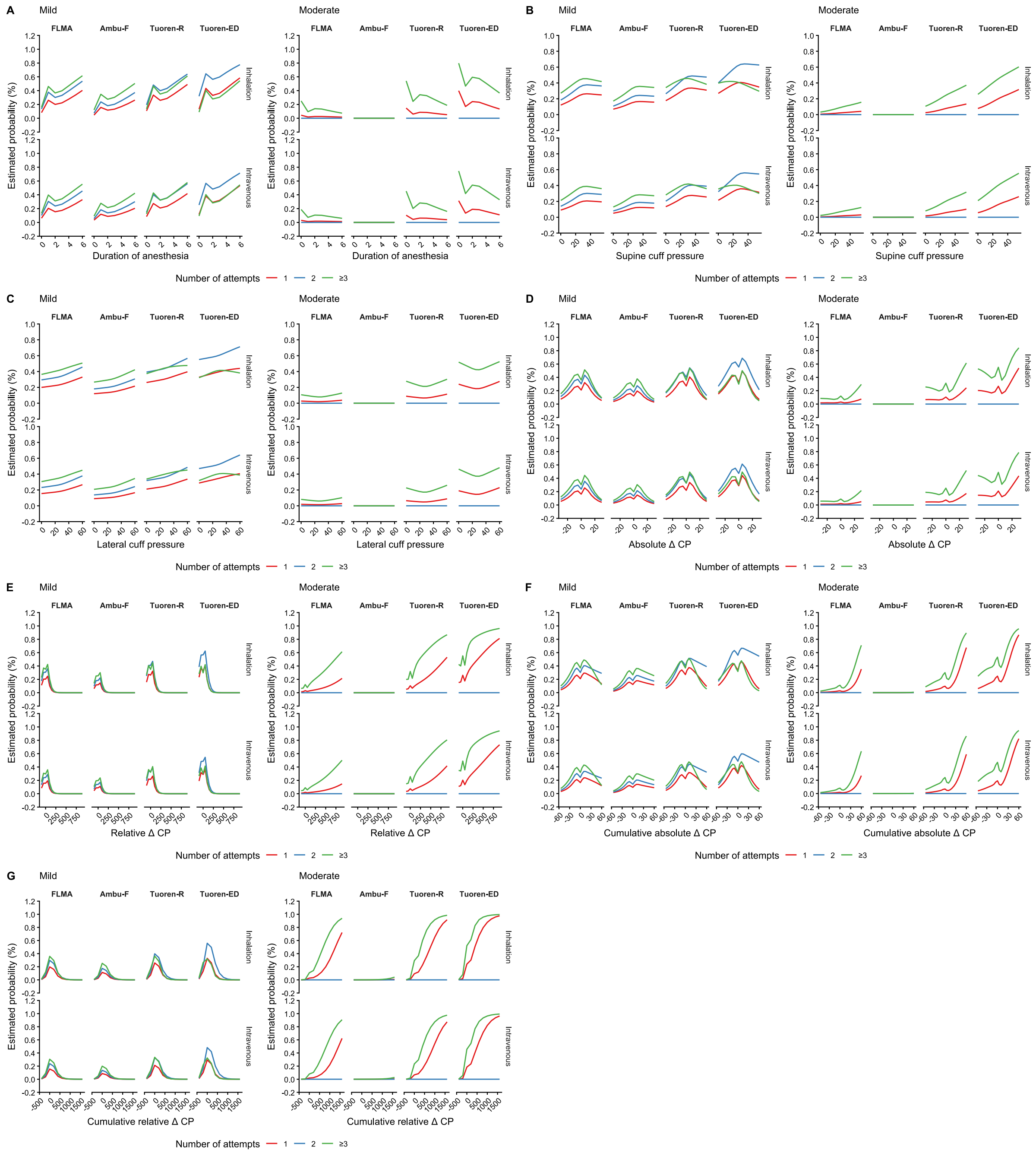

Figure S3. Adjusted predicted probabilities of POST severity across duration of anesthesia and intracuff pressure metrics based on 50 imputations

(A) Duration of anesthesia. (B) Supine cuff pressure. (C) Lateral cuff pressure. (D) Absolute change in CP. (E) Relative change in CP. (F) Cumulative absolute change in CP. (G) Cumulative relative change in CP.

Adjusted probabilities were estimated using the multinomial models, in which duration of anesthesia, supine and lateral cuff pressure, absolute and relative change in CP, and cumulative absolute and relative change in CP were modeled with restricted cubic splines.

Ambu-F, Ambu AuraFlex; CP, cuff pressure; FLMA, laryngeal mask airway Flexible; LMA, laryngeal mask airway; POST, postoperative sore throat; Tuoren-ED, Tuoren Esophagus Drainage Type I; Tuoren-R, Tuoren Reinforced Type.

Table S1. Potential factors associated with acute POST in patients undergoing elective tympanoplasty

Variables	Definition	Collection
**Preoperative factors**		
Age	Age at admission for tympanoplasty	At admission
Sex	Sex at admission for tympanoplasty	At admission
Smoking	≥1 cigarette per day	At admission
Weight	Measured during physical examinations	At admission
Height	Measured during physical examinations	At admission
BMI	The weight in kilograms divided by the square of the height in meters	Passive
**Intraoperative factors**		
Type of LMA	Chosen by anesthetists	Intraoperative
Size of LMA	Chosen by anesthetists, often standardized by sex	Intraoperative
Anesthetic method	The way of maintaining the status of general anesthesia	Intraoperative
Surgery ear	The ear which required surgical operations	Intraoperative
Number of attempts	The insertion number until correctly placing the LMA with an adequate seal	Intraoperative
Duration of anesthesia	The time from anesthesia induction up to awake extubation	Intraoperative
Supine cuff pressure	Measured at the supine, neutral position	Intraoperative
Lateral cuff pressure	Measured after adjusting patients' positions and turning their heads contralateral to the operative ear	Intraoperative
Δ in CP	Subtracting the supine cuff pressure from the lateral cuff pressure	Passive
Δ% in CP	Dividing the change of CP by supine cuff pressure	Passive
Cumulative Δ in CP	Defined as multiplying the absolute change in CP by the duration of anesthesia	Passive
Cumulative Δ% in CP	Multiplying the relative change in CP by the duration of anesthesia	Passive

BMI, body mass index; CP, cuff pressure; LMA, laryngeal mask airway; POST, postoperative sore throat; Δ, absolute change; Δ%, relative change.

Table S2. Missing numbers and rates of demographic and intraoperative factors

Variables	Missing number	Missing rate
**Total number (N=1,363)**		
Age	0	0
Female	0	0
Smoking	704	51.50%
Weight	0	0
Height	0	0
BMI	0	0
Type of LMA	0	0
Size of LMA	0	0
Anesthetic method	618	45.21%
Surgery ear	468	34.24%
Number of attempts	0	0
Duration of anesthesia	15	1.10%
Supine cuff pressure	1	0.07%
Lateral cuff pressure	1	0.07%
Absolute change in CP	1	0.07%
Relative change in CP	1	0.07%
Cumulative absolute change in CP	16	1.17%
Cumulative relative change in CP	16	1.17%

BMI, body mass index; LMA, laryngeal mask airway; POST, postoperative sore throat; Tuoren-ED, Tuoren Esophagus Drainage Type I; Tuoren-R, Tuoren Reinforced Type.

Table S3. Pooled clinical characteristics of patients by POST severity

Variables, N (%)	POST severity		
	**No (N = 823)**	**Mild (N = 470)**	**Moderate-to-severe (N = 70)**
Age, years	44.01 ± 13.52	44.97 ± 13.06	46.27 ± 12.88
Female	427 (51.9)	296 (63.0)	53 (75.7)
Smoking	106 (12.9)	46 (9.7)	4 (6.4)
Weight, kg	63.17 ± 11.45	62.86 ± 12.22	60.71 ± 9.62
Height, cm	165.37 ± 7.91	163.94 ± 8.00	162.71 ± 6.98
BMI, kg/m^2	23.00 ± 3.20	23.26 ± 3.37	22.88 ± 2.97
Type of LMA			
FLMA	432 (52.5)	162 (34.5)	8 (11.4)
Ambu-F	28 (3.4)	7 (1.5)	
Tuoren-R	119 (14.5)	70 (14.9)	9 (12.9)
Tuoren-ED	244 (29.6)	231 (49.1)	53 (75.7)
Size of LMA			
3	415 (50.4)	281 (59.8)	54 (77.1)
4 or 5	408 (49.6)	189 (40.2)	16 (22.9)
Anesthetic method			
Inhalation	123 (15.0)	82 (17.5)	20 (28.5)
Intravenous	700 (85.0)	388 (82.5)	50 (71.5)
Surgery ear			
Right	390 (47.4)	240 (51.0)	39 (54.9)
Left	419 (51.0)	222 (47.2)	30 (42.8)
Both	14 (1.7)	8 (1.8)	2 (2.3)
Number of attempts			
1	780 (94.8)	433 (92.1)	67 (95.7)
2	36 (4.4)	29 (6.2)	
3	7 (0.9)	8 (1.7)	3 (4.3)
Duration of anesthesia, h	1.28 [0.95, 1.82]	1.28 [0.98, 1.72]	1.38 [0.94, 1.75]
Intracuff pressure, cmH_2O			
Supine	27.38 ± 8.79	28.56 ± 8.52	30.50 ± 8.99
Lateral	26.71 ± 9.10	28.42 ± 9.18	30.29 ± 9.15
Absolute Δ	0.00 [-4.00, 4.00]	0.00 [-4.00, 4.13]	0.00 [-2.00, 4.00]
Relative Δ	0.00 [-17.80, 18.02]	0.00 [-15.43, 20.00]	0.00 [-9.84, 10.83]
Cumulative absolute Δ	0.00 [-6.47, 4.96]	0.00 [-6.14, 5.61]	0.00 [-3.95, 4.89]
Cumulative relative Δ	0.00 [-22.67, 20.45]	0.00 [-21.05, 24.10]	0.00 [-13.91, 17.35]

Ambu-F, Ambu AuraFlex; BMI, body mass index; FLMA, LMA Flexible; LMA, laryngeal mask airway; POST, postoperative sore throat; Tuoren-ED, Tuoren Esophagus Drainage Type I; Tuoren-R, Tuoren Reinforced Type.

For continuous variables with normality, we calculated the pooled mean (standard deviation) based on Rubin's rules, and median [interquartile range, IQR] and frequencies (percentages) were pooled as the average values across 50 imputed datasets.

Absolute and relative Δ refer to absolute and relative change in CP, whereas cumulative absolute and relative Δ refer to cumulative absolute and relative change in CP, respectively.

Table S4. Univariate regression for POST incidence and severity based on 50 imputations

Factors	POST incidence		POST severity			
			Mild pain		Moderate-to-severe pain	
	OR (95% CI)	*P* value	OR (95% CI)	*P* value	OR (95% CI)	*P* value
Type of LMA						
FLMA	1.000 (Ref)		1.000 (Ref)		1.000 (Ref)	
Ambu-F	0.635 (0.272, 1.483)	0.294	0.666 (0.285, 1.556)	0.348	N.A.	0.710
Tuoren-R	1.687 (1.206, 2.360)	0.002	1.569 (1.109, 2.218)	0.011	4.088 (1.543, 10.831)	0.005
Tuoren-ED	2.958 (2.311, 3.785)	<0.001	2.525 (1.957, 3.259)	<0.001	11.730 (5.484, 25.093)	<0.001
Size of LMA						
3	1.000 (Ref)		1.000 (Ref)		1.000 (Ref)	
4 or 5	0.622 (0.499, 0.777)	<0.001	0.684 (0.544, 0.861)	0.001	0.301 (0.170, 0.536)	<0.001
Anesthetic method						
Inhalation	1.000 (Ref)		1.000 (Ref)		N.A.	
intravenous	0.758 (0.497, 1.157)	0.197	0.833 (0.561, 1.236)	0.362	0.455 (0.166, 1.242)	0.123
Number of attempts						
1	1.000 (Ref)		1.000 (Ref)		1.000 (Ref)	
2	1.257 (0.761, 2.077)	0.372	1.450 (0.877, 2.399)	0.148	N.A.	0.638
3	2.451 (0.943, 6.371)	0.066	2.053 (0.739, 5.701)	0.167	4.944 (1.245, 19.632)	0.023
Duration of anesthesia, h	1.028 (0.886, 1.193)	0.712	1.028 (0.880, 1.201)	0.726	1.031 (0.738, 1.440)	0.860
<0.88	1.000 (Ref)		1.000 (Ref)		1.000 (Ref)	
0.88 to 1.15	1.295 (0.910, 1.842)	0.151	1.394 (0.963, 2.018)	0.078	0.790 (0.352, 1.772)	0.566
1.15 to 1.45	1.284 (0.904, 1.824)	0.162	1.384 (0.959, 1.997)	0.083	0.779 (0.347, 1.751)	0.546
1.45 to 1.98	1.331 (0.939, 1.887)	0.108	1.362 (0.943, 1.967)	0.100	1.177 (0.568, 2.439)	0.662
≥1.98	1.021 (0.715, 1.458)	0.908	1.045 (0.717, 1.522)	0.819	0.901 (0.422, 1.924)	0.788
Supine cuff pressure, cmH_2O	1.019 (1.006, 1.032)	0.003	1.016 (1.003, 1.029)	0.019	1.042 (1.013, 1.071)	0.004
<20	1.000 (Ref)		1.000 (Ref)		1.000 (Ref)	
20 to 24	1.061 (0.711, 1.585)	0.770	1.155 (0.763, 1.749)	0.496	0.418 (0.123, 1.418)	0.161
24 to 30	1.534 (1.055, 2.230)	0.025	1.566 (1.060, 2.315)	0.024	1.310 (0.534, 3.213)	0.555
30 to 37	1.963 (1.358, 2.837)	<0.001	1.833 (1.244, 2.701)	0.002	2.854 (1.264, 6.447)	0.012
≥37	1.450 (0.987, 2.128)	0.058	1.434 (0.959, 2.144)	0.079	1.557 (0.640, 3.784)	0.328
Lateral cuff pressure, cmH_2O	1.024 (1.011, 1.036)	<0.001	1.021 (1.008, 1.034)	0.001	1.044 (1.016, 1.072)	0.002
<20	1.000 (Ref)		1.000 (Ref)		1.000 (Ref)	
20 to 25	1.154 (0.805, 1.656)	0.435	1.100 (0.755, 1.602)	0.621	1.643 (0.679, 3.978)	0.271

25 to 30	1.547 (1.065, 2.245)	0.022	1.568 (1.067, 2.304)	0.022	1.358 (0.510, 3.620)	0.540
30 to 36	1.274 (0.895, 1.813)	0.179	1.263 (0.875, 1.821)	0.212	1.372 (0.555, 3.395)	0.493
≥36	1.923 (1.346, 2.746)	<0.001	1.746 (1.203, 2.535)	0.003	3.489 (1.525, 7.982)	0.003
Absolute change in CP, cmH_2O	1.010 (0.995, 1.025)	0.195	1.010 (0.994, 1.026)	0.207	1.009 (0.975, 1.044)	0.611
<-6	1.000 (Ref)		1.000 (Ref)		1.000 (Ref)	
-6 to -2	1.042 (0.715, 1.520)	0.829	1.063 (0.720, 1.572)	0.757	0.871 (0.327, 2.321)	0.783
-2 to 1	1.241 (0.877, 1.757)	0.223	1.116 (0.774, 1.608)	0.556	2.258 (1.030, 4.947)	0.042
1 to 6	1.326 (0.920, 1.910)	0.130	1.313 (0.899, 1.919)	0.158	1.425 (0.591, 3.437)	0.430
≥6	1.314 (0.922, 1.874)	0.131	1.323 (0.916, 1.911)	0.136	1.244 (0.517, 2.995)	0.626
Relative change in CP, %	1.000 (0.997, 1.003)	0.968	1.000 (0.997, 1.003)	0.940	1.000 (0.994, 1.006)	0.925
<-21.40	1.000 (Ref)		1.000 (Ref)		1.000 (Ref)	
-21.40 to -7.14	1.055 (0.743, 1.498)	0.764	1.029 (0.715, 1.479)	0.879	1.310 (0.537, 3.193)	0.552
-7.14 to 4.55	1.107 (0.782, 1.568)	0.565	0.962 (0.667, 1.389)	0.838	2.511 (1.128, 5.589)	0.024
4.55 to 25.00	1.463 (1.034, 2.072)	0.032	1.430 (0.998, 2.048)	0.051	1.790 (0.752, 4.260)	0.188
≥25.00	1.153 (0.814, 1.633)	0.422	1.134 (0.791, 1.625)	0.494	1.341 (0.550, 3.270)	0.518
Cumulative absolute change in CP, cmH_2O	1.010 (1.000, 1.020)	0.053	1.009 (0.999, 1.019)	0.079	1.013 (0.991, 1.035)	0.244
<-7.83	1.000 (Ref)		1.000 (Ref)		1.000 (Ref)	
-7.83 to 2.53	1.163 (0.822, 1.646)	0.394	1.223 (0.853, 1.755)	0.273	0.762 (0.318, 1.824)	0.541
-2.53 to 1.20	1.012 (0.713, 1.437)	0.945	0.905 (0.623, 1.315)	0.600	1.725 (0.842, 3.533)	0.136
1.20 to 6.77	1.192 (0.843, 1.685)	0.321	1.236 (0.862, 1.773)	0.248	0.895 (0.388, 2.061)	0.794
≥6.77	1.315 (0.931, 1.856)	0.120	1.333 (0.931, 1.911)	0.117	1.191 (0.542, 2.614)	0.663
Cumulative relative change in CP, %	1.000 (0.999, 1.002)	0.589	1.000 (0.999, 1.002)	0.685	1.001 (0.998, 1.004)	0.520
<-28.40	1.000 (Ref)		1.000 (Ref)		1.000 (Ref)	
-28.40 to -9.17	1.052 (0.743, 1.490)	0.775	1.021 (0.711, 1.465)	0.912	1.341 (0.572, 3.145)	0.500
-9.17 to 4.0	0.988 (0.697, 1.401)	0.948	0.860 (0.594, 1.244)	0.423	2.169 (0.996, 4.725)	0.051
4.0 to 28.8	1.303 (0.924, 1.838)	0.131	1.276 (0.894, 1.821)	0.179	1.553 (0.669, 3.606)	0.306
≥28.8	1.162 (0.823, 1.641)	0.393	1.162 (0.814, 1.660)	0.408	1.162 (0.480, 2.812)	0.739

Ambu-F, Ambu AuraFlex; BMI, body mass index; CP, cuff pressure; FLMA, LMA Flexible; LMA, laryngeal mask airway; POST, postoperative sore throat; Tuoren-ED, Tuoren Esophagus Drainage Type I; Tuoren-R, Tuoren Reinforced Type.

Univariate logistic and multinomial log-linear models were respectively used to estimate crude ORs for acute incident POST and POST severity, in which continuous variables were also categorized based on their quintiles. The quintiles of each variable were determined by stacking all completed datasets. All results were pooled based on Rubin’s rules.

Table S5. Variance Inflation Factor for each intraoperative factor and its confounders

Exposure factors	Model variables	VIF values
Type of LMA	Type of LMA	1.036<br>1.174<br>1.399
	Age	1.068
	Age2	1.037
	Female	1.287
	Smoking	1.194
	BMI	1.301
Size of LMA	Size of LMA	5.087
	Age	1.054
	Age2	1.025
	Female	5.049
	Smoking	1.203
	BMI	1.156
Anesthetic method	Anesthetic method	1.004
	Age	1.054
	Age2	1.025
	Female	1.265
	Smoking	1.194
	BMI	1.143
Number of attempts	Number of attempts	1.013<br>1.020
	Age	1.069
	Age2	1.040
	Female	5.074
	Smoking	1.209
	BMI	1.324
	Type of LMA	1.043<br>1.178<br>1.409
	Size of LMA	5.119
Duration of anesthesia	Duration of anesthesia	1.024
	Age	1.070
	Age2	1.044
	Female	5.074
	Smoking	1.212
	BMI	1.332
	Type of LMA	1.044<br>1.179<br>1.414
	Size of LMA	5.121
	Number of attempts	1.013<br>1.026
	Anesthetic method	1.019
Supine cuff pressure	Supine cuff pressure	1.012
	Age	1.071
	Age2	1.045
	Female	5.078
	Smoking	1.212
	BMI	1.330
	Type of LMA	1.044<br>1.179

		1.414
	Size of LMA	5.129
	Number of attempts	1.013<br>1.023
	Anesthetic method	1.011
Lateral cuff pressure	Lateral cuff pressure	1.932
	Age	1.074
	Age2	1.048
	Female	5.086
	Smoking	1.213
	BMI	1.335
	Type of LMA	1.049<br>1.181<br>1.412
	Size of LMA	5.125
	Number of attempts	1.014<br>1.024
	Anesthetic method	1.011
	Supine cuff pressure	1.896
Absolute change in CP	Absolute change in CP	1.185
	Age	1.074
	Age2	1.048
	Female	5.086
	Smoking	1.213
	BMI	1.335
	Type of LMA	1.049<br>1.181<br>1.412
	Size of LMA	5.125
	Number of attempts	1.014<br>1.024
	Anesthetic method	1.011
	Supine cuff pressure	1.161
Relative change in CP	Relative change in CP	1.135
	Age	1.073
	Age2	1.046
	Female	5.091
	Smoking	1.213
	BMI	1.332
	Type of LMA	1.047<br>1.181<br>1.413
	Size of LMA	5.131
	Number of attempts	1.014<br>1.024
	Anesthetic method	1.011
	Supine cuff pressure	1.126
Cumulative absolute change in CP	Cumulative absolute change in CP	1.146
	Age	1.072
	Age2	1.049
	Female	5.081
	Smoking	1.213
	BMI	1.334
	Type of LMA	1.047

		1.183<br>1.410
	Size of LMA	5.119
	Number of attempts	1.015<br>1.024
	Anesthetic method	1.012
	Supine cuff pressure	1.132
Cumulative relative change in CP	Cumulative relative change in CP	1.104
	Age	1.072
	Age2	1.047
	Female	5.090
	Smoking	1.215
	BMI	1.331
	Type of LMA	1.046<br>1.182<br>1.414
	Size of LMA	5.131
	Number of attempts	1.015<br>1.024
	Anesthetic method	1.012
	Supine cuff pressure	1.102

The multicollinearity diagnostic results are very similar across 50 imputed data sets, so we used the results from the first completed dataset.

For multiple categorical variables, VIF values were calculated for their dummy variables, including Ambu-F, Tuoren-R, Tuoren-ED for type of LMA, and number of attempts of 2, and number of attempts of ≥3 for number of insertion attempts.

Table S6. Associations between quintiles of duration of anesthesia and intracuff pressure metrics with POST incidence and severity based on 50 imputations

Factors	POST incidence			POST severity					
				Mild			Moderate-to-severe		
	OR (95% CI)	*P* value	*P*	OR (95% CI)	*P* value	*P*	OR (95% CI)	*P* value	*P*
Duration of anesthesia			0.953			0.941			0.874
<0.88	1.000 (Ref)			1.000 (Ref)			1.000 (Ref)		
0.88 to 1.15	1.390 (0.958, 2.017)	0.083		1.473 (1.003, 2.163)	0.048		0.827 (0.344, 1.990)	0.672	
1.15 to 1.45	1.280 (0.883, 1.854)	0.192		1.375 (0.939, 2.015)	0.102		0.670 (0.280, 1.600)	0.367	
1.45 to 1.98	1.348 (0.932, 1.951)	0.113		1.384 (0.943, 2.030)	0.097		1.131 (0.513, 2.493)	0.760	
≥1.98	1.010 (0.693, 1.471)	0.959		1.028 (0.695, 1.521)	0.889		0.930 (0.405, 2.136)	0.864	
Supine cuff pressure			0.003			0.011			0.019
<20	1.000 (Ref)			1.000 (Ref)			1.000 (Ref)		
20 to 24	1.173 (0.769, 1.789)	0.459		1.248 (0.810, 1.922)	0.316		0.472 (0.132, 1.683)	0.247	
24 to 30	1.545 (1.041, 2.291)	0.031		1.572 (1.047, 2.361)	0.029		1.291 (0.497, 3.352)	0.599	
**30 to 37**	1.837 (1.244, 2.712)	0.002		1.744 (1.164, 2.614)	0.007		2.458 (1.027, 5.884)	0.043	
≥37	1.572 (1.048, 2.361)	0.029		1.556 (1.023, 2.366)	0.039		1.528 (0.584, 4.000)	0.387	
Lateral cuff pressure			0.461			0.436			0.905
<20	1.000 (Ref)			1.000 (Ref)			1.000 (Ref)		
20 to 25	1.053 (0.713, 1.554)	0.796		1.027 (0.687, 1.534)	0.898		1.220 (0.464, 3.203)	0.687	
25 to 30	1.266 (0.831, 1.929)	0.272		1.320 (0.858, 2.029)	0.206		0.800 (0.267, 2.398)	0.690	
30 to 36	0.990 (0.645, 1.518)	0.962		1.025 (0.661, 1.589)	0.912		0.653 (0.219, 1.940)	0.442	
≥36	1.316 (0.803, 2.155)	0.276		1.296 (0.780, 2.153)	0.317		1.278 (0.394, 4.146)	0.682	
Absolute change in CP			0.078			0.083			0.670
<-6	1.000 (Ref)			1.000 (Ref)			1.000 (Ref)		
-6 to -2	0.905 (0.605, 1.355)	0.628		0.933 (0.618, 1.409)	0.743		0.661 (0.231, 1.885)	0.438	
-2 to 1	1.074 (0.737, 1.565)	0.711		1.001 (0.678, 1.477)	0.998		1.663 (0.695, 3.980)	0.253	
1 to 6	1.190 (0.795, 1.782)	0.397		1.198 (0.793, 1.810)	0.390		1.062 (0.397, 2.842)	0.904	
≥6	1.290 (0.864, 1.925)	0.213		1.300 (0.864, 1.959)	0.208		1.079 (0.399, 2.913)	0.881	
Relative change in CP (%)			0.147			0.176			0.546
<-21.40	1.000 (Ref)			1.000 (Ref)			1.000 (Ref)		
-21.40 to -7.14	0.920 (0.636, 1.332)	0.660		0.912 (0.625, 1.331)	0.632		1.039 (0.403, 2.683)	0.937	
-7.14 to 4.55	0.876 (0.603, 1.271)	0.484		0.798 (0.542, 1.174)	0.251		1.647 (0.689, 3.934)	0.261	

4.55 to 25.00	1.184 (0.815, 1.721)	0.376		1.187 (0.811, 1.737)	0.378		1.129 (0.440, 2.896)	0.800	
≥25.00	1.227 (0.833, 1.807)	0.301		1.205 (0.811, 1.789)	0.355		1.364 (0.504, 3.688)	0.540	
Cumulative absolute change in CP			0.107			0.117			0.640
<-7.83	1.000 (Ref)			1.000 (Ref)			1.000 (Ref)		
-7.83 to 2.53	1.158 (0.800, 1.677)	0.437		1.204 (0.824, 1.759)	0.336		0.776 (0.306, 1.969)	0.593	
-2.53 to 1.20	0.979 (0.672, 1.426)	0.910		0.896 (0.604, 1.328)	0.584		1.569 (0.707, 3.483)	0.268	
1.20 to 6.77	1.179 (0.805, 1.726)	0.397		1.218 (0.824, 1.799)	0.322		0.793 (0.315, 1.997)	0.622	
≥6.77	1.425 (0.966, 2.101)	0.074		1.431 (0.961, 2.132)	0.078		1.263 (0.516, 3.092)	0.608	
Cumulative relative change in CP			0.158			0.169			0.704
<-28.40	1.000 (Ref)			1.000 (Ref)			1.000 (Ref)		
-28.40 to -9.17	0.951 (0.659, 1.374)	0.791		0.930 (0.639, 1.355)	0.707		1.266 (0.507, 3.159)	0.613	
-9.17 to 4.0	0.839 (0.578, 1.216)	0.353		0.757 (0.514, 1.114)	0.158		1.697 (0.727, 3.963)	0.221	
4.0 to 28.8	1.156 (0.800, 1.671)	0.439		1.151 (0.791, 1.675)	0.463		1.162 (0.466, 2.902)	0.747	
≥28.8	1.262 (0.860, 1.851)	0.234		1.255 (0.850, 1.855)	0.253		1.264 (0.477, 3.348)	0.637	

CP, cuff pressure; POST, postoperative sore throat.

All logistic and multinomial models were adjusted for age, age squared, female sex, smoking, and BMI. Additionally, each model was further adjusted for type of LMA, size of LMA, anesthetic method, and number of attempts for duration of anesthesia and supine cuff pressure, and were adjusted for type of LMA, size of LMA, anesthetic method, number of attempts, and supine cuff pressure for lateral cuff pressure, position-induced absolute and relative change in CP, and cumulative absolute and relative change in CP. P for trend was calculated by modeling categorized duration of anesthesia and intracuff pressure metrics as continuous variables. The quintiles of each variable were determined by stacking all completed datasets. All results were pooled based on Rubin’s rules.

Table S7. Adjusted odds ratios for associations between intraoperative factors and POST incidence and severity in the overlap weighting population based on 50 imputations

Factors	POST incidence		POST severity			
			Mild pain		Moderate-to-severe pain	
	OR (95% CI)	*P* value	OR (95% CI)	*P* value	OR (95% CI)	*P* value
Type of LMA						
FLMA	1.000 (Ref)		1.000 (Ref)		1.000 (Ref)	
Ambu-F	0.521 (0.221, 1.230)	0.137	0.550 (0.233, 1.301)	0.174	0.000 (0.000, 0.000)	<0.001
Tuoren-R	1.936 (1.295, 2.894)	0.001	1.734 (1.143, 2.630)	0.010	5.497 (1.858, 16.260)	0.002
Tuoren-ED	3.632 (2.564, 5.144)	<0.001	2.955 (2.058, 4.243)	<0.001	15.622 (6.364, 38.346)	<0.001
Size of LMA						
3	1.000 (Ref)		1.000 (Ref)		1.000 (Ref)	
4 or 5	0.948 (0.571, 1.575)	0.837	1.092 (0.649, 1.839)	0.740	0.295 (0.073, 1.189)	0.086
Anesthetic method						
Inhalation	1.000 (Ref)		1.000 (Ref)		1.000 (Ref)	
intravenous	0.779 (0.514, 1.182)	0.239	0.855 (0.578, 1.265)	0.432	0.474 (0.177, 1.269)	0.136
Number of attempts						
1	1.000 (Ref)		1.000 (Ref)		1.000 (Ref)	
2	1.683 (0.910, 3.112)	0.097	1.939 (1.046, 3.594)	0.035	0.000 (0.000, 0.000)	<0.001
3	2.996 (1.113, 8.065)	0.030	2.435 (0.847, 7.000)	0.098	6.690 (1.601, 27.960)	0.009
Duration of anesthesia	1.002 (0.856, 1.174)	0.978	1.002 (0.849, 1.181)	0.983	1.003 (0.701, 1.437)	0.985
<0.88	1.000 (Ref)		1.000 (Ref)		1.000 (Ref)	
0.88 to 1.15	1.353 (0.945, 1.936)	0.098	1.451 (0.997, 2.111)	0.052	0.882 (0.383, 2.032)	0.769
1.15 to 1.45	1.270 (0.890, 1.813)	0.188	1.380 (0.951, 2.002)	0.090	0.744 (0.326, 1.697)	0.482
1.45 to 1.98	1.309 (0.917, 1.867)	0.138	1.358 (0.934, 1.975)	0.109	1.071 (0.506, 2.270)	0.857
≥1.98	0.977 (0.676, 1.411)	0.900	1.002 (0.680, 1.476)	0.993	0.856 (0.388, 1.888)	0.700
Supine cuff pressure	1.017 (1.004, 1.030)	0.010	1.014 (1.001, 1.028)	0.036	1.035 (1.006, 1.065)	0.016
<20	1.000 (Ref)		1.000 (Ref)		1.000 (Ref)	
20 to 24	1.267 (0.829, 1.936)	0.273	1.380 (0.889, 2.141)	0.151	0.554 (0.155, 1.973)	0.362
24 to 30	1.656 (1.116, 2.459)	0.012	1.715 (1.134, 2.592)	0.011	1.288 (0.504, 3.292)	0.597
30 to 37	1.935 (1.306, 2.867)	<0.001	1.830 (1.210, 2.768)	0.004	2.606 (1.101, 6.172)	0.029
≥37	1.697 (1.126, 2.557)	0.011	1.724 (1.122, 2.647)	0.013	1.530 (0.580, 4.032)	0.390
Lateral cuff pressure	0.998 (0.969, 1.028)	0.904	0.995 (0.965, 1.027)	0.766	1.018 (0.978, 1.061)	0.379
<20	1.000 (Ref)		1.000 (Ref)		1.000 (Ref)	
20 to 25	1.557 (0.871, 2.784)	0.135	1.430 (0.780, 2.622)	0.248	2.915 (0.687, 12.369)	0.147
25 to 30	1.594 (0.890, 2.855)	0.117	1.601 (0.874, 2.930)	0.127	1.528 (0.338, 6.903)	0.582
30 to 36	1.428 (0.798, 2.558)	0.230	1.408 (0.769, 2.578)	0.268	1.652 (0.362, 7.536)	0.517

≥36	1.333 (0.664, 2.675)	0.419	1.275 (0.614, 2.645)	0.515	1.950 (0.425, 8.943)	0.390
Absolute change in CP	1.016 (0.995, 1.038)	0.127	1.016 (0.995, 1.038)	0.139	1.016 (0.980, 1.052)	0.390
<-6	1.000 (Ref)		1.000 (Ref)		1.000 (Ref)	
-6 to -2	0.853 (0.555, 1.311)	0.469	0.896 (0.573, 1.401)	0.631	0.555 (0.187, 1.652)	0.291
-2 to 1	1.081 (0.722, 1.618)	0.704	1.004 (0.656, 1.536)	0.987	1.619 (0.682, 3.839)	0.275
1 to 6	1.219 (0.798, 1.862)	0.361	1.234 (0.793, 1.921)	0.352	1.111 (0.419, 2.945)	0.832
≥6	1.296 (0.844, 1.991)	0.236	1.326 (0.848, 2.074)	0.217	1.091 (0.408, 2.918)	0.862
Relative change in CP	1.000 (0.994, 1.006)	0.944	1.000 (0.993, 1.006)	0.899	1.001 (0.994, 1.009)	0.741
<-21.40	1.000 (Ref)		1.000 (Ref)		1.000 (Ref)	
-21.40 to -7.14	0.917 (0.626, 1.343)	0.657	0.905 (0.609, 1.343)	0.619	1.036 (0.393, 2.731)	0.944
-7.14 to 4.55	0.878 (0.598, 1.290)	0.508	0.772 (0.514, 1.160)	0.213	1.874 (0.792, 4.434)	0.153
4.55 to 25.00	1.188 (0.816, 1.729)	0.369	1.183 (0.802, 1.744)	0.397	1.233 (0.490, 3.103)	0.657
≥25.00	1.261 (0.832, 1.911)	0.274	1.220 (0.793, 1.879)	0.365	1.645 (0.605, 4.476)	0.330
Cumulative absolute change in CP	1.021 (1.007, 1.034)	0.002	1.018 (1.005, 1.032)	0.007	1.036 (0.994, 1.080)	0.094
<-7.83	1.000 (Ref)		1.000 (Ref)		1.000 (Ref)	
-7.83 to 2.53	1.215 (0.826, 1.786)	0.323	1.240 (0.831, 1.850)	0.292	1.003 (0.388, 2.596)	0.994
-2.53 to 1.20	1.076 (0.730, 1.587)	0.711	0.944 (0.625, 1.426)	0.783	2.168 (0.961, 4.892)	0.062
1.20 to 6.77	1.252 (0.849, 1.847)	0.257	1.267 (0.846, 1.896)	0.251	1.131 (0.444, 2.886)	0.796
≥6.77	1.649 (1.100, 2.470)	0.015	1.604 (1.052, 2.443)	0.028	2.021 (0.818, 4.990)	0.127
Cumulative relative change in CP	1.002 (0.999, 1.006)	0.243	1.002 (0.998, 1.005)	0.371	1.005 (0.997, 1.012)	0.212
<-28.40	1.000 (Ref)		1.000 (Ref)		1.000 (Ref)	
-28.40 to -9.17	0.931 (0.639, 1.356)	0.708	0.904 (0.612, 1.336)	0.612	1.181 (0.467, 2.990)	0.725
-9.17 to 4.0	0.830 (0.571, 1.206)	0.329	0.718 (0.483, 1.068)	0.102	1.877 (0.821, 4.294)	0.136
4.0 to 28.8	1.109 (0.773, 1.591)	0.575	1.099 (0.757, 1.598)	0.619	1.197 (0.499, 2.872)	0.687
≥28.8	1.396 (0.935, 2.086)	0.103	1.398 (0.923, 2.117)	0.114	1.378 (0.509, 3.733)	0.528

Ambu-F, Ambu AuraFlex; FLMA, LMA Flexible; LMA, laryngeal mask airway; N.A., not available; POST, postoperative sore throat; OW, overlap weighting; Tuoren-ED, Tuoren Esophagus Drainage Type I; Tuoren-R, Tuoren Reinforced Type.

Propensity score was calculated by the overlap weighting method. All models were adjusted for age, age squared, female sex, smoking, and BMI. Additionally, each model was further adjusted for type of LMA, size of LMA, anesthetic method, and number of attempts for duration of anesthesia and supine cuff pressure, and were adjusted for type of LMA, size of LMA, anesthetic method, number of attempts, and supine cuff pressure for lateral cuff pressure, position-induced absolute and relative change in CP, and cumulative absolute and relative change in CP. The quintiles of each variable were determined by stacking all completed datasets. All results were pooled based on Rubin’s rules.

Table S8. Age-stratified adjusted odds ratios for associations between intraoperative factors and acute POST incidence and severity based on 50 imputations

**Factors**	**POST incidence**			**POST severity**				***P***
				**Mild**		**Moderate-to-severe**		
	**< 50 y**	**≥ 50 y**	***P***	**< 50 y**	**≥ 50 y**	**< 50 y**	**≥ 50 y**	
Type of LMA			0.874					0.602
FLMA	1.000 (Ref)	1.000 (Ref)		1.000 (Ref)	1.000 (Ref)	1.000 (Ref)	1.000 (Ref)	
Ambu-F	0.424 (0.138, 1.305)	0.643 (0.171, 2.417)		0.469 (0.152, 1.447)	0.662 (0.176, 2.496)	0.000 (NaN, NaN)	0.000 (0.000, 0.001)	
Tuoren-R	1.703 (1.121, 2.586)	1.651 (0.907, 3.007)		1.680 (1.097, 2.573)	1.350 (0.718, 2.538)	2.069 (0.583, 7.341)	17.637 (1.970, 157.937)	
Tuoren-ED	3.242 (2.283, 4.603)	3.746 (2.410, 5.823)		2.783 (1.937, 4.000)	2.904 (1.842, 4.577)	11.419 (4.651, 28.034)	52.501 (6.825, 403.837)	
Size of LMA			0.897					0.574
3	1.000 (Ref)	1.000 (Ref)		1.000 (Ref)	1.000 (Ref)	1.000 (Ref)	1.000 (Ref)	
4 or 5	0.792 (0.396, 1.583)	1.093 (0.515, 2.320)		0.957 (0.469, 1.955)	1.209 (0.551, 2.653)	0.133 (0.021, 0.851)	0.602 (0.109, 3.312)	
Anesthetic method			0.478					0.666
Inhalation	1.000 (Ref)	1.000 (Ref)		1.000 (Ref)	1.000 (Ref)	1.000 (Ref)	1.000 (Ref)	
Intravenous	0.818 (0.494, 1.355)	0.698 (0.363, 1.339)		0.866 (0.532, 1.410)	0.809 (0.412, 1.588)	0.582 (0.151, 2.247)	0.368 (0.101, 1.345)	
Number of attempts			0.872					0.952
1	1.000 (Ref)	1.000 (Ref)		1.000 (Ref)	1.000 (Ref)	1.000 (Ref)	1.000 (Ref)	
2	1.697 (0.860, 3.349)	1.329 (0.560, 3.151)		1.882 (0.957, 3.703)	1.465 (0.621, 3.455)	0.000 (NaN, NaN)	0.000 (0.000, 0.000)	
3	3.918 (0.894, 17.171)	2.969 (0.676, 13.039)		3.614 (0.803, 16.266)	2.355 (0.461, 12.040)	6.236 (0.449, 86.581)	11.339 (0.939, 136.979)	
Duration of anesthesia	0.976 (0.794, 1.200)	1.085 (0.842, 1.400)	0.604	0.997 (0.808, 1.231)	1.056 (0.811, 1.375)	0.791 (0.450, 1.391)	1.421 (0.845, 2.390)	0.622
<0.88	1.000 (Ref)	1.000 (Ref)	0.884	1.000 (Ref)	1.000 (Ref)	1.000 (Ref)	1.000 (Ref)	0.618
0.88 to 1.15	1.408 (0.868, 2.286)	1.379 (0.761, 2.501)		1.441 (0.875, 2.375)	1.543 (0.836, 2.847)	1.029 (0.325, 3.264)	0.494 (0.106, 2.293)	
1.15 to 1.45	1.202 (0.744, 1.944)	1.373 (0.752, 2.506)		1.272 (0.776, 2.085)	1.500 (0.805, 2.797)	0.644 (0.192, 2.162)	0.694 (0.183, 2.631)	
1.45 to 1.98	1.494 (0.928, 2.408)	1.084 (0.595, 1.976)		1.462 (0.892, 2.398)	1.213 (0.650, 2.261)	1.579 (0.550, 4.534)	0.504 (0.128, 1.977)	
≥1.98	0.879 (0.533, 1.448)	1.243 (0.689, 2.244)		0.944 (0.565, 1.577)	1.193 (0.639, 2.228)	0.430 (0.111, 1.671)	1.758 (0.566, 5.459)	
Supine cuff pressure	1.019 (1.001,	1.020 (0.998,	0.938	1.016 (0.999,	1.017 (0.994,	1.039 (0.997,	1.036 (0.988,	0.970

	1.036)	1.042)		1.034)	1.040)	1.084)	1.086)	
<20	1.000 (Ref)	1.000 (Ref)	0.936	1.000 (Ref)	1.000 (Ref)	1.000 (Ref)	1.000 (Ref)	0.994
20 to 24	0.997 (0.579, 1.715)	1.529 (0.766, 3.050)		1.117 (0.642, 1.943)	1.529 (0.751, 3.116)	0.128 (0.014, 1.207)	1.292 (0.226, 7.371)	
24 to 30	1.312 (0.800, 2.149)	2.114 (1.082, 4.131)		1.360 (0.817, 2.265)	2.116 (1.061, 4.219)	0.931 (0.276, 3.149)	1.936 (0.394, 9.511)	
30 to 37	1.685 (1.025, 2.769)	2.203 (1.158, 4.191)		1.595 (0.951, 2.675)	2.068 (1.060, 4.032)	2.223 (0.722, 6.843)	3.181 (0.763, 13.267)	
≥37	1.445 (0.866, 2.411)	1.845 (0.931, 3.655)		1.496 (0.883, 2.535)	1.686 (0.828, 3.432)	0.927 (0.253, 3.389)	2.955 (0.630, 13.865)	
Lateral cuff pressure	1.009 (0.987, 1.032)	1.017 (0.987, 1.047)	0.705	1.010 (0.987, 1.033)	1.015 (0.984, 1.046)	1.000 (0.946, 1.057)	1.031 (0.964, 1.103)	0.939
<20	1.000 (Ref)	1.000 (Ref)	0.207	1.000 (Ref)	1.000 (Ref)	1.000 (Ref)	1.000 (Ref)	0.578
20 to 25	1.089 (0.662, 1.790)	0.962 (0.506, 1.830)		1.083 (0.649, 1.807)	0.909 (0.467, 1.769)	1.008 (0.285, 3.571)	1.551 (0.326, 7.385)	
25 to 30	1.027 (0.593, 1.779)	1.709 (0.866, 3.373)		1.100 (0.628, 1.927)	1.701 (0.850, 3.405)	0.431 (0.095, 1.956)	2.092 (0.369, 11.862)	
30 to 36	0.952 (0.555, 1.634)	1.038 (0.503, 2.142)		0.993 (0.571, 1.728)	1.059 (0.505, 2.223)	0.559 (0.138, 2.271)	0.939 (0.152, 5.799)	
≥36	1.266 (0.670, 2.392)	1.422 (0.635, 3.186)		1.294 (0.672, 2.490)	1.317 (0.573, 3.026)	0.924 (0.195, 4.390)	2.659 (0.394, 17.927)	
Absolute change in CP	1.009 (0.987, 1.032)	1.017 (0.987, 1.047)	0.502	1.010 (0.987, 1.033)	1.015 (0.984, 1.046)	1.000 (0.946, 1.057)	1.031 (0.964, 1.103)	0.555
<-6	1.000 (Ref)	1.000 (Ref)	0.865	1.000 (Ref)	1.000 (Ref)	1.000 (Ref)	1.000 (Ref)	0.936
-6 to -2	1.105 (0.659, 1.852)	0.632 (0.323, 1.236)		1.192 (0.702, 2.024)	0.600 (0.302, 1.191)	0.460 (0.114, 1.857)	1.172 (0.210, 6.559)	
-2 to 1	1.092 (0.672, 1.774)	1.035 (0.556, 1.927)		1.072 (0.647, 1.775)	0.888 (0.469, 1.683)	1.074 (0.359, 3.217)	4.226 (0.905, 19.734)	
1 to 6	1.474 (0.879, 2.472)	0.814 (0.417, 1.592)		1.547 (0.909, 2.633)	0.765 (0.386, 1.513)	0.927 (0.268, 3.210)	1.654 (0.307, 8.915)	
≥6	1.377 (0.820, 2.312)	1.193 (0.615, 2.312)		1.418 (0.831, 2.421)	1.153 (0.591, 2.251)	0.950 (0.277, 3.258)	1.781 (0.340, 9.328)	
Relative change in CP	1.000 (0.996, 1.004)	1.003 (0.995, 1.010)	0.884	1.000 (0.995, 1.004)	1.002 (0.995, 1.010)	1.001 (0.988, 1.014)	1.005 (0.988, 1.023)	0.992
<-21.40	1.000 (Ref)	1.000 (Ref)	0.262	1.000 (Ref)	1.000 (Ref)	1.000 (Ref)	1.000 (Ref)	0.713
-21.40 to -7.14	0.828 (0.515, 1.330)	1.110 (0.602, 2.046)		0.847 (0.521, 1.375)	1.042 (0.558, 1.945)	0.630 (0.181, 2.198)	2.625 (0.565, 12.202)	
-7.14 to 4.55	0.832 (0.515, 1.344)	0.944 (0.512, 1.743)		0.782 (0.475, 1.287)	0.811 (0.430, 1.531)	1.146 (0.378, 3.475)	3.823 (0.847, 17.255)	

4.55 to 25.00	1.456 (0.904, 2.344)	0.821 (0.437, 1.540)		1.497 (0.921, 2.434)	0.778 (0.410, 1.478)	1.064 (0.326, 3.473)	1.594 (0.323, 7.874)	
≥25.00	1.050 (0.637, 1.732)	1.668 (0.876, 3.175)		1.035 (0.619, 1.730)	1.608 (0.839, 3.082)	1.096 (0.314, 3.823)	2.760 (0.548, 13.905)	
Cumulative absolute change in CP	1.011 (0.997, 1.025)	1.015 (0.996, 1.034)	0.373	1.011 (0.996, 1.026)	1.012 (0.993, 1.032)	1.008 (0.973, 1.045)	1.040 (0.997, 1.086)	0.292
<-7.83	1.000 (Ref)	1.000 (Ref)	0.321	1.000 (Ref)	1.000 (Ref)	1.000 (Ref)	1.000 (Ref)	0.700
-7.83 to 2.53	1.509 (0.937, 2.429)	0.745 (0.402, 1.383)		1.591 (0.977, 2.590)	0.746 (0.396, 1.403)	0.847 (0.243, 2.946)	0.723 (0.165, 3.177)	
-2.53 to 1.20	1.014 (0.622, 1.651)	0.927 (0.500, 1.719)		0.953 (0.572, 1.587)	0.807 (0.424, 1.539)	1.355 (0.464, 3.953)	2.468 (0.677, 8.995)	
1.20 to 6.77	1.656 (1.016, 2.699)	0.673 (0.356, 1.273)		1.709 (1.035, 2.823)	0.686 (0.358, 1.314)	1.139 (0.348, 3.726)	0.530 (0.112, 2.516)	
≥6.77	1.468 (0.889, 2.425)	1.420 (0.743, 2.712)		1.487 (0.887, 2.495)	1.400 (0.725, 2.704)	1.263 (0.389, 4.098)	1.519 (0.373, 6.185)	
Cumulative relative change in CP	1.001 (0.998, 1.003)	1.003 (0.998, 1.007)	0.770	1.000 (0.998, 1.003)	1.002 (0.998, 1.007)	1.003 (0.996, 1.009)	1.009 (0.997, 1.020)	0.711
<-28.40	1.000 (Ref)	1.000 (Ref)	0.091	1.000 (Ref)	1.000 (Ref)	1.000 (Ref)	1.000 (Ref)	0.347
-28.40 to -9.17	0.925 (0.578, 1.481)	1.027 (0.559, 1.886)		0.901 (0.556, 1.458)	1.001 (0.537, 1.865)	1.300 (0.378, 4.469)	1.264 (0.296, 5.404)	
-9.17 to 4.0	0.857 (0.531, 1.382)	0.811 (0.440, 1.493)		0.780 (0.475, 1.280)	0.713 (0.377, 1.351)	1.693 (0.530, 5.410)	2.067 (0.551, 7.747)	
4.0 to 28.8	1.471 (0.917, 2.361)	0.814 (0.443, 1.496)		1.456 (0.900, 2.357)	0.803 (0.431, 1.497)	1.639 (0.480, 5.598)	0.849 (0.204, 3.533)	
≥28.8	1.073 (0.654, 1.759)	1.754 (0.928, 3.315)		1.068 (0.645, 1.769)	1.744 (0.915, 3.324)	1.134 (0.303, 4.248)	1.779 (0.409, 7.741)	

Ambu-F, Ambu AuraFlex; CP, cuff pressure; FLMA, laryngeal mask airway Flexible; LMA, laryngeal mask airway; N.A., not available; POST, postoperative sore throat; Tuoren-ED, Tuoren Esophagus Drainage Type I; Tuoren-R, Tuoren Reinforced Type.

All logistic and multinomial models were adjusted for age, age squared, female sex, smoking, and BMI. Additionally, each model was further adjusted for type of LMA, size of LMA, anesthetic method, and number of attempts for duration of anesthesia and supine cuff pressure, and were adjusted for type of LMA, size of LMA, anesthetic method, number of attempts, and supine cuff pressure for lateral cuff pressure, position-induced absolute and relative change in CP, and cumulative absolute and relative change in CP. P for interaction was calculated by comparing the full logistic or multinomial models with and without the age-interaction term *via* likelihood ratio test. The quintiles of each variable were determined by stacking all completed datasets. All results were pooled based on Rubin’s rules.

Table S9. Sex-stratified adjusted odds ratios for associations between intraoperative factors and acute POST incidence and severity based on 50 imputations

Factors	POST incidence			POST severity				
				Mild		Moderate-to-severe		*P*
	Male	Female	*P*	Male	Female	Male	Female	
Type of LMA			0.248					0.349
FLMA	1.000 (Ref)	1.000 (Ref)		1.000 (Ref)	1.000 (Ref)	1.000 (Ref)	1.000 (Ref)	
Ambu-F	0.000 (0.000, Inf)	0.580 (0.244, 1.382)		0.000 (0.000, 0.000)	0.611 (0.256, 1.456)	0.000 (0.000, 0.001)	0.000 (0.000, 0.000)	
Tuoren-R	1.377 (0.768, 2.468)	1.831 (1.202, 2.789)		1.419 (0.790, 2.548)	1.630 (1.052, 2.525)	0.000 (0.000, 0.000)	5.392 (1.842, 15.783)	
Tuoren-ED	2.529 (1.657, 3.859)	4.211 (2.935, 6.043)		2.177 (1.408, 3.365)	3.429 (2.360, 4.984)	14.190 (3.015, 66.777)	18.608 (7.398, 46.805)	
Size of LMA			0.289					0.636
3	1.000 (Ref)	1.000 (Ref)		1.000 (Ref)	1.000 (Ref)	1.000 (Ref)	1.000 (Ref)	
4 or 5	0.598 (0.243, 1.472)	1.128 (0.613, 2.076)		0.691 (0.263, 1.817)	1.291 (0.697, 2.393)	0.279 (0.050, 1.569)	0.295 (0.037, 2.328)	
Anesthetic method			0.517					0.683
Inhalation	1.000 (Ref)	1.000 (Ref)		1.000 (Ref)	1.000 (Ref)	1.000 (Ref)	1.000 (Ref)	
Intravenous	0.870 (0.444, 1.704)	0.730 (0.446, 1.193)		0.932 (0.481, 1.803)	0.813 (0.502, 1.316)	0.523 (0.081, 3.363)	0.444 (0.155, 1.274)	
Number of attempts			0.468					0.355
1	1.000 (Ref)	1.000 (Ref)		1.000 (Ref)	1.000 (Ref)	1.000 (Ref)	1.000 (Ref)	
2	1.092 (0.470, 2.533)	2.060 (1.015, 4.181)		1.163 (0.503, 2.691)	2.309 (1.142, 4.666)	0.000 (0.000, 0.000)	0.000 (0.000, 0.000)	
3	2.732 (0.640, 11.660)	4.137 (0.790, 21.650)		2.961 (0.699, 12.551)	2.930 (0.501, 17.147)	0.000 (0.000, 0.000)	16.275 (1.964, 134.843)	
Duration of anesthesia	0.913 (0.722, 1.154)	1.143 (0.914, 1.429)	0.471	0.911 (0.716, 1.158)	1.141 (0.907, 1.436)	0.941 (0.476, 1.864)	1.164 (0.743, 1.825)	0.825
<0.88	1.000 (Ref)	1.000 (Ref)	0.070	1.000 (Ref)	1.000 (Ref)	1.000 (Ref)	1.000 (Ref)	0.324
0.88 to 1.15	1.087 (0.617, 1.915)	1.720 (1.038, 2.849)		1.153 (0.647, 2.057)	1.832 (1.084, 3.093)	0.534 (0.087, 3.278)	1.063 (0.370, 3.052)	
1.15 to 1.45	1.002 (0.565, 1.776)	1.561 (0.949, 2.566)		1.031 (0.572, 1.858)	1.716 (1.025, 2.871)	0.782 (0.158, 3.863)	0.785 (0.273, 2.263)	
1.45 to 1.98	1.418 (0.810, 2.480)	1.352 (0.821, 2.225)		1.426 (0.802, 2.535)	1.402 (0.833, 2.359)	1.489 (0.341, 6.513)	1.087 (0.413, 2.860)	
≥1.98	0.619 (0.349, 1.098)	1.569 (0.932, 2.640)		0.634 (0.350, 1.145)	1.607 (0.933, 2.768)	0.520 (0.107, 2.523)	1.371 (0.493, 3.808)	
Supine cuff pressure	1.019 (0.998,	1.016 (0.998,	0.744	1.017 (0.996,	1.014 (0.995,	1.040 (0.980,	1.031 (0.993,	0.926

	1.041)	1.034)		1.039)	1.032)	1.103)	1.069)	
<20	1.000 (Ref)	1.000 (Ref)	0.453	1.000 (Ref)	1.000 (Ref)	1.000 (Ref)	1.000 (Ref)	0.788
20 to 24	0.827 (0.433, 1.578)	1.447 (0.821, 2.552)		0.907 (0.469, 1.756)	1.517 (0.848, 2.712)	0.182 (0.016, 2.050)	0.743 (0.158, 3.481)	
24 to 30	0.964 (0.520, 1.786)	2.068 (1.228, 3.482)		1.030 (0.547, 1.940)	2.068 (1.206, 3.546)	0.440 (0.065, 2.988)	2.119 (0.663, 6.769)	
30 to 37	1.393 (0.769, 2.523)	2.164 (1.284, 3.644)		1.339 (0.723, 2.481)	2.037 (1.184, 3.505)	1.913 (0.431, 8.494)	3.140 (1.042, 9.465)	
≥37	1.264 (0.678, 2.357)	1.676 (0.972, 2.890)		1.305 (0.688, 2.476)	1.646 (0.936, 2.897)	0.755 (0.122, 4.684)	1.750 (0.529, 5.790)	
Lateral cuff pressure	0.996 (0.971, 1.023)	1.023 (0.998, 1.048)	0.394	0.995 (0.969, 1.022)	1.024 (0.999, 1.050)	1.016 (0.935, 1.104)	1.012 (0.961, 1.065)	0.664
<20	1.000 (Ref)	1.000 (Ref)	0.347	1.000 (Ref)	1.000 (Ref)	1.000 (Ref)	1.000 (Ref)	0.268
20 to 25	0.815 (0.467, 1.421)	1.308 (0.749, 2.284)		0.783 (0.442, 1.387)	1.309 (0.734, 2.335)	1.329 (0.263, 6.717)	1.252 (0.364, 4.307)	
25 to 30	1.136 (0.610, 2.115)	1.401 (0.780, 2.516)		1.199 (0.640, 2.248)	1.477 (0.807, 2.704)	0.393 (0.032, 4.750)	0.932 (0.249, 3.479)	
30 to 36	0.518 (0.260, 1.032)	1.409 (0.786, 2.524)		0.478 (0.233, 0.977)	1.538 (0.843, 2.805)	1.004 (0.140, 7.215)	0.640 (0.165, 2.479)	
≥36	0.940 (0.454, 1.948)	1.684 (0.849, 3.341)		0.949 (0.452, 1.995)	1.666 (0.819, 3.390)	0.921 (0.087, 9.716)	1.548 (0.368, 6.503)	
Absolute change in CP	0.996 (0.971, 1.023)	1.023 (0.998, 1.048)	0.562	0.995 (0.969, 1.022)	1.024 (0.999, 1.050)	1.016 (0.935, 1.104)	1.012 (0.961, 1.065)	0.826
<-6	1.000 (Ref)	1.000 (Ref)	0.461	1.000 (Ref)	1.000 (Ref)	1.000 (Ref)	1.000 (Ref)	0.604
-6 to -2	1.074 (0.600, 1.920)	0.777 (0.439, 1.376)		1.052 (0.582, 1.899)	0.839 (0.467, 1.510)	1.361 (0.190, 9.754)	0.410 (0.110, 1.528)	
-2 to 1	1.140 (0.663, 1.959)	1.000 (0.586, 1.705)		1.044 (0.599, 1.820)	0.950 (0.544, 1.659)	3.360 (0.579, 19.487)	1.181 (0.412, 3.385)	
1 to 6	1.117 (0.618, 2.017)	1.206 (0.685, 2.125)		1.111 (0.610, 2.021)	1.240 (0.691, 2.225)	1.195 (0.142, 10.076)	0.974 (0.305, 3.105)	
≥6	0.935 (0.500, 1.747)	1.456 (0.838, 2.530)		0.874 (0.460, 1.660)	1.552 (0.879, 2.741)	2.033 (0.268, 15.397)	0.843 (0.258, 2.758)	
Relative change in CP	0.998 (0.991, 1.004)	1.003 (0.997, 1.008)	0.517	0.997 (0.990, 1.004)	1.003 (0.997, 1.009)	1.003 (0.991, 1.014)	1.001 (0.989, 1.014)	0.753
<-21.40	1.000 (Ref)	1.000 (Ref)	0.216	1.000 (Ref)	1.000 (Ref)	1.000 (Ref)	1.000 (Ref)	0.301
-21.40 to -7.14	1.088 (0.645, 1.836)	0.747 (0.439, 1.271)		1.044 (0.613, 1.778)	0.760 (0.440, 1.310)	2.297 (0.375, 14.055)	0.660 (0.200, 2.178)	
-7.14 to 4.55	0.822 (0.470, 1.438)	0.886 (0.531, 1.478)		0.748 (0.420, 1.331)	0.810 (0.475, 1.379)	2.555 (0.423, 15.443)	1.364 (0.483, 3.855)	

4.55 to 25.00	1.177 (0.672, 2.062)	1.142 (0.683, 1.908)		1.183 (0.672, 2.082)	1.146 (0.677, 1.942)	1.050 (0.120, 9.178)	1.109 (0.371, 3.316)	
≥25.00	0.765 (0.402, 1.454)	1.513 (0.903, 2.535)		0.679 (0.349, 1.321)	1.560 (0.920, 2.644)	3.394 (0.451, 25.529)	1.105 (0.341, 3.584)	
Cumulative absolute change in CP	1.006 (0.990, 1.022)	1.017 (1.001, 1.033)	0.647	1.004 (0.988, 1.021)	1.017 (1.000, 1.033)	1.028 (0.978, 1.079)	1.017 (0.983, 1.052)	0.819
<-7.83	1.000 (Ref)	1.000 (Ref)	0.644	1.000 (Ref)	1.000 (Ref)	1.000 (Ref)	1.000 (Ref)	0.769
-7.83 to 2.53	1.153 (0.669, 1.987)	1.218 (0.726, 2.043)		1.250 (0.720, 2.170)	1.243 (0.730, 2.118)	0.236 (0.024, 2.294)	1.049 (0.342, 3.221)	
-2.53 to 1.20	0.932 (0.541, 1.607)	1.023 (0.602, 1.741)		0.881 (0.500, 1.553)	0.918 (0.525, 1.605)	1.583 (0.396, 6.327)	1.635 (0.594, 4.500)	
1.20 to 6.77	0.831 (0.464, 1.487)	1.466 (0.864, 2.489)		0.843 (0.463, 1.534)	1.532 (0.890, 2.635)	0.702 (0.134, 3.675)	0.976 (0.308, 3.095)	
≥6.77	1.262 (0.694, 2.296)	1.596 (0.939, 2.711)		1.279 (0.694, 2.356)	1.609 (0.932, 2.777)	1.039 (0.166, 6.520)	1.466 (0.491, 4.378)	
Cumulative relative change in CP	1.000 (0.997, 1.003)	1.003 (1.000, 1.007)	0.697	0.999 (0.996, 1.003)	1.003 (0.999, 1.007)	1.002 (0.997, 1.008)	1.005 (0.996, 1.013)	0.822
<-28.40	1.000 (Ref)	1.000 (Ref)	0.340	1.000 (Ref)	1.000 (Ref)	1.000 (Ref)	1.000 (Ref)	0.386
-28.40 to -9.17	1.052 (0.617, 1.795)	0.862 (0.515, 1.441)		1.037 (0.601, 1.790)	0.841 (0.496, 1.425)	1.538 (0.291, 8.117)	1.109 (0.357, 3.449)	
-9.17 to 4.0	0.989 (0.575, 1.702)	0.712 (0.424, 1.195)		0.916 (0.523, 1.603)	0.626 (0.364, 1.077)	2.227 (0.460, 10.779)	1.398 (0.492, 3.970)	
4.0 to 28.8	0.842 (0.472, 1.501)	1.331 (0.807, 2.196)		0.887 (0.495, 1.589)	1.302 (0.781, 2.172)	0.279 (0.023, 3.430)	1.599 (0.545, 4.693)	
≥28.8	1.197 (0.654, 2.192)	1.279 (0.767, 2.134)		1.122 (0.604, 2.086)	1.313 (0.779, 2.211)	2.806 (0.449, 17.538)	1.024 (0.313, 3.347)	

Ambu-F, Ambu AuraFlex; CP, cuff pressure; FLMA, laryngeal mask airway Flexible; LMA, laryngeal mask airway; N.A., not available; POST, postoperative sore throat; Tuoren-ED, Tuoren Esophagus Drainage Type I; Tuoren-R, Tuoren Reinforced Type.

All logistic and multinomial models were adjusted for age, age squared, female sex, smoking, and BMI. Additionally, each model was further adjusted for type of LMA, size of LMA, anesthetic method, and number of attempts for duration of anesthesia and supine cuff pressure, and were adjusted for type of LMA, size of LMA, anesthetic method, number of attempts, and supine cuff pressure for lateral cuff pressure, position-induced absolute and relative change in CP, and cumulative absolute and relative change in CP. P for interaction was calculated by comparing the full logistic or multinomial models with and without the sex-interaction term *via* likelihood ratio test. The quintiles of each variable were determined by stacking all completed datasets. All results were pooled based on Rubin’s rules.

Table S10. Overweight-stratified adjusted odds ratios for associations between intraoperative factors and acute POST incidence and severity based on 50 imputations

Factors	POST incidence			POST severity				
				Mild		Moderate-to-severe		
	< 24 kg/m²	≥ 24 kg/m²	***P***	< 24 kg/m²	≥ 24 kg/m²	< 24 kg/m²	≥ 24 kg/m²	***P***
Type of LMA			0.088					0.287
FLMA	1.000 (Ref)	1.000 (Ref)		1.000 (Ref)	1.000 (Ref)	1.000 (Ref)	1.000 (Ref)	
Ambu-F	0.849 (0.341, 2.116)	0.000 (0.000, Inf)		0.907 (0.364, 2.264)	0.000 (0.000, 0.000)	0.000 (0.000, 0.001)	0.000 (0.000, 0.000)	
Tuoren-R	1.823 (1.239, 2.681)	1.342 (0.632, 2.848)		1.687 (1.134, 2.512)	1.298 (0.602, 2.800)	4.777 (1.601, 14.253)	1.941 (0.165, 22.774)	
Tuoren-ED	3.310 (2.360, 4.642)	3.653 (2.232, 5.977)		2.698 (1.897, 3.839)	3.082 (1.859, 5.110)	16.427 (6.542, 41.250)	16.654 (3.614, 76.741)	
Size of LMA			0.457					0.648
3	1.000 (Ref)	1.000 (Ref)		1.000 (Ref)	1.000 (Ref)	1.000 (Ref)	1.000 (Ref)	
4 or 5	0.702 (0.380, 1.298)	1.802 (0.688, 4.720)		0.863 (0.456, 1.631)	1.882 (0.698, 5.077)	0.131 (0.026, 0.672)	1.440 (0.197, 10.545)	
Anesthetic method			0.497					0.708
Inhalation	1.000 (Ref)	1.000 (Ref)		1.000 (Ref)	1.000 (Ref)	1.000 (Ref)	1.000 (Ref)	
Intravenous	0.824 (0.503, 1.348)	0.681 (0.339, 1.369)		0.903 (0.559, 1.459)	0.747 (0.367, 1.522)	0.508 (0.155, 1.669)	0.404 (0.102, 1.608)	
Number of attempts			0.657					0.587
1	1.000 (Ref)	1.000 (Ref)		1.000 (Ref)	1.000 (Ref)	1.000 (Ref)	1.000 (Ref)	
2	1.611 (0.863, 3.006)	1.297 (0.459, 3.668)		1.790 (0.961, 3.334)	1.428 (0.512, 3.982)	0.000 (NaN, NaN)	0.000 (0.000, 0.000)	
3	4.274 (1.290, 14.160)	1.365 (0.195, 9.550)		4.137 (1.224, 13.986)	0.604 (0.048, 7.633)	3.842 (0.281, 52.523)	4.874 (0.436, 54.504)	
Duration of anesthesia	1.094 (0.891, 1.344)	0.908 (0.701, 1.175)	0.631	1.089 (0.882, 1.346)	0.912 (0.700, 1.187)	1.197 (0.731, 1.959)	0.851 (0.453, 1.601)	0.915
<0.88	1.000 (Ref)	1.000 (Ref)	0.825	1.000 (Ref)	1.000 (Ref)	1.000 (Ref)	1.000 (Ref)	0.521
0.88 to 1.15	1.437 (0.899, 2.298)	1.399 (0.742, 2.636)		1.456 (0.896, 2.364)	1.585 (0.826, 3.041)	1.288 (0.430, 3.855)	0.340 (0.060, 1.925)	
1.15 to 1.45	1.444 (0.904, 2.305)	1.123 (0.602, 2.093)		1.501 (0.928, 2.429)	1.283 (0.674, 2.440)	0.962 (0.319, 2.901)	0.381 (0.083, 1.758)	
1.45 to 1.98	1.322 (0.829, 2.109)	1.450 (0.775, 2.715)		1.386 (0.857, 2.241)	1.439 (0.744, 2.781)	0.860 (0.275, 2.686)	1.245 (0.369, 4.206)	
≥1.98	1.160 (0.712, 1.891)	0.819 (0.443, 1.514)		1.102 (0.661, 1.838)	0.928 (0.491, 1.754)	1.729 (0.583, 5.130)	0.261 (0.057, 1.199)	
Supine cuff	1.013	1.034	0.248	1.010	1.033	1.036	1.042	0.445

pressure	(0.996, 1.030)	(1.010, 1.059)		(0.993, 1.027)	(1.007, 1.058)	(0.997, 1.076)	(0.987, 1.100)	
<20	1.000 (Ref)	1.000 (Ref)	0.563	1.000 (Ref)	1.000 (Ref)	1.000 (Ref)	1.000 (Ref)	0.376
20 to 24	1.124 (0.673, 1.876)	1.436 (0.650, 3.173)		1.189 (0.706, 2.002)	1.639 (0.715, 3.756)	0.389 (0.070, 2.157)	0.489 (0.068, 3.510)	
24 to 30	1.416 (0.881, 2.276)	2.098 (0.999, 4.407)		1.422 (0.874, 2.314)	2.285 (1.045, 4.996)	1.292 (0.388, 4.300)	1.276 (0.263, 6.179)	
30 to 37	1.773 (1.100, 2.857)	2.364 (1.143, 4.890)		1.597 (0.973, 2.620)	2.509 (1.166, 5.402)	3.329 (1.138, 9.736)	1.636 (0.360, 7.433)	
≥37	1.334 (0.814, 2.185)	2.423 (1.125, 5.219)		1.287 (0.774, 2.140)	2.618 (1.171, 5.853)	1.610 (0.483, 5.366)	1.307 (0.247, 6.921)	
Lateral cuff pressure	1.018 (0.997, 1.041)	0.993 (0.962, 1.025)	0.947	1.019 (0.997, 1.042)	0.993 (0.961, 1.025)	1.009 (0.957, 1.063)	1.007 (0.933, 1.087)	0.989
<20	1.000 (Ref)	1.000 (Ref)	0.177	1.000 (Ref)	1.000 (Ref)	1.000 (Ref)	1.000 (Ref)	0.254
20 to 25	1.176 (0.728, 1.901)	0.884 (0.441, 1.772)		1.206 (0.737, 1.975)	0.790 (0.384, 1.623)	0.765 (0.220, 2.653)	2.040 (0.360, 11.548)	
25 to 30	1.094 (0.645, 1.853)	1.370 (0.653, 2.875)		1.122 (0.652, 1.931)	1.445 (0.681, 3.068)	0.770 (0.202, 2.934)	0.762 (0.101, 5.736)	
30 to 36	1.178 (0.703, 1.974)	0.649 (0.294, 1.433)		1.232 (0.725, 2.094)	0.678 (0.302, 1.520)	0.639 (0.171, 2.382)	0.548 (0.067, 4.473)	
≥36	1.722 (0.935, 3.172)	0.700 (0.291, 1.684)		1.725 (0.920, 3.236)	0.684 (0.278, 1.683)	1.278 (0.299, 5.459)	1.033 (0.120, 8.913)	
Absolute change in CP	1.018 (0.997, 1.041)	0.993 (0.962, 1.025)	0.498	1.019 (0.997, 1.042)	0.993 (0.961, 1.025)	1.009 (0.957, 1.063)	1.007 (0.933, 1.087)	0.680
<-6	1.000 (Ref)	1.000 (Ref)	0.363	1.000 (Ref)	1.000 (Ref)	1.000 (Ref)	1.000 (Ref)	0.319
-6 to -2	0.887 (0.532, 1.480)	0.922 (0.463, 1.837)		0.955 (0.567, 1.610)	0.925 (0.458, 1.867)	0.342 (0.076, 1.549)	0.849 (0.154, 4.687)	
-2 to 1	1.123 (0.695, 1.816)	0.978 (0.520, 1.843)		1.000 (0.605, 1.653)	0.974 (0.511, 1.858)	1.986 (0.673, 5.863)	1.112 (0.234, 5.282)	
1 to 6	1.440 (0.870, 2.383)	0.784 (0.392, 1.567)		1.482 (0.885, 2.480)	0.775 (0.381, 1.576)	0.932 (0.262, 3.319)	0.990 (0.191, 5.123)	
≥6	1.501 (0.917, 2.456)	0.896 (0.430, 1.865)		1.545 (0.933, 2.558)	0.899 (0.426, 1.899)	1.019 (0.300, 3.458)	0.969 (0.164, 5.734)	
Relative change in CP	1.001 (0.997, 1.004)	0.996 (0.989, 1.004)	0.734	1.001 (0.997, 1.004)	0.996 (0.988, 1.004)	1.000 (0.987, 1.013)	1.003 (0.984, 1.022)	0.710
<-21.40	1.000 (Ref)	1.000 (Ref)	0.273	1.000 (Ref)	1.000 (Ref)	1.000 (Ref)	1.000 (Ref)	0.141
-21.40 to -7.14	0.729 (0.456, 1.166)	1.313 (0.695, 2.482)		0.740 (0.458, 1.194)	1.293 (0.675, 2.476)	0.579 (0.160, 2.097)	1.598 (0.325, 7.872)	
-7.14 to 4.55	0.796 (0.496,	0.974 (0.516,		0.665 (0.403,	0.983 (0.514,	2.044 (0.702,	1.021 (0.205,	

	1.278)	1.839)		1.097)	1.880)	5.950)	5.082)	
4.55 to 25.00	1.277 (0.804, 2.029)	0.997 (0.516, 1.925)		1.277 (0.796, 2.046)	1.029 (0.526, 2.013)	1.125 (0.347, 3.644)	0.907 (0.173, 4.761)	
≥25.00	1.259 (0.793, 1.998)	0.993 (0.469, 2.105)		1.272 (0.795, 2.036)	0.908 (0.418, 1.973)	0.997 (0.290, 3.426)	2.324 (0.404, 13.362)	
Cumulative absolute change in CP	1.015 (1.001, 1.029)	1.005 (0.986, 1.025)	0.293	1.015 (1.000, 1.030)	1.004 (0.984, 1.024)	1.014 (0.979, 1.050)	1.026 (0.982, 1.073)	0.523
<-7.83	1.000 (Ref)	1.000 (Ref)	0.322	1.000 (Ref)	1.000 (Ref)	1.000 (Ref)	1.000 (Ref)	0.128
-7.83 to 2.53	1.342 (0.837, 2.151)	0.942 (0.505, 1.757)		1.423 (0.878, 2.306)	0.955 (0.505, 1.806)	0.651 (0.179, 2.365)	0.822 (0.195, 3.463)	
-2.53 to 1.20	0.984 (0.603, 1.606)	0.934 (0.503, 1.733)		0.830 (0.491, 1.404)	0.937 (0.497, 1.765)	2.098 (0.769, 5.723)	0.972 (0.241, 3.918)	
1.20 to 6.77	1.557 (0.961, 2.521)	0.688 (0.360, 1.316)		1.673 (1.023, 2.735)	0.657 (0.336, 1.285)	0.503 (0.132, 1.922)	1.052 (0.266, 4.154)	
≥6.77	1.638 (1.016, 2.641)	1.071 (0.528, 2.171)		1.644 (1.005, 2.689)	1.120 (0.545, 2.301)	1.502 (0.505, 4.464)	0.738 (0.140, 3.904)	
Cumulative relative change in CP	1.001 (0.999, 1.003)	1.000 (0.996, 1.005)	0.532	1.001 (0.999, 1.003)	1.000 (0.995, 1.004)	1.003 (0.996, 1.009)	1.007 (0.995, 1.018)	0.649
<-28.40	1.000 (Ref)	1.000 (Ref)	0.149	1.000 (Ref)	1.000 (Ref)	1.000 (Ref)	1.000 (Ref)	0.209
-28.40 to -9.17	0.887 (0.554, 1.419)	1.048 (0.567, 1.938)		0.890 (0.550, 1.440)	0.984 (0.526, 1.842)	0.840 (0.253, 2.794)	2.263 (0.469, 10.927)	
-9.17 to 4.0	0.729 (0.452, 1.176)	0.988 (0.530, 1.842)		0.627 (0.377, 1.040)	0.933 (0.494, 1.762)	1.685 (0.597, 4.756)	2.050 (0.432, 9.723)	
4.0 to 28.8	1.390 (0.881, 2.192)	0.753 (0.394, 1.442)		1.392 (0.874, 2.216)	0.750 (0.388, 1.452)	1.201 (0.402, 3.586)	1.117 (0.203, 6.166)	
≥28.8	1.255 (0.782, 2.016)	1.164 (0.586, 2.312)		1.287 (0.795, 2.086)	1.108 (0.550, 2.232)	0.855 (0.247, 2.956)	2.172 (0.414, 11.405)	

Ambu-F, Ambu AuraFlex; CP, cuff pressure; FLMA, laryngeal mask airway Flexible; LMA, laryngeal mask airway; N.A., not available; POST, postoperative sore throat; Tuoren-ED, Tuoren Esophagus Drainage Type I; Tuoren-R, Tuoren Reinforced Type.

All logistic and multinomial models were adjusted for age, age squared, female sex, smoking, and BMI. Additionally, each model was further adjusted for type of LMA, size of LMA, anesthetic method, and number of attempts for duration of anesthesia and supine cuff pressure, and were adjusted for type of LMA, size of LMA, anesthetic method, number of attempts, and supine cuff pressure for lateral cuff pressure, position-induced absolute and relative change in CP, and cumulative absolute and relative change in CP. P for interaction was calculated by comparing the full logistic or multinomial models with and without the BMI-interaction term *via* likelihood ratio test. The quintiles of each variable were determined by stacking all completed datasets. All results were pooled based on Rubin’s rules.

Table S11. Smoking-stratified adjusted odds ratios for associations between intraoperative factors and acute POST incidence and severity based on 50 imputations

**Factors**	**POST incidence**			**POST severity**				
				**Mild**		**Moderate-to-severe**		***P***
	Non-smoker	Smoker	***P***	Non-smoker	Smoker	Non-smoker	Smoker	
Type of LMA			0.498					0.615
FLMA	1.000 (Ref)	1.000 (Ref)		1.000 (Ref)	1.000 (Ref)	1.000 (Ref)	1.000 (Ref)	
Ambu-F	0.520 (0.221, 1.224)			0.541 (0.230, 1.273)	1.000 (1.000, 1.000)	0.000 (0.000, 0.000)	1.000 (NaN, NaN)	
Tuoren-R	1.867 (1.300, 2.679)	0.507 (0.100, 2.573)		1.691 (1.177, 2.429)	1.422 (0.766, 2.639)	4.236 (1.585, 11.324)	12.110 (3.298, 44.467)	
Tuoren-ED	3.636 (2.692, 4.910)	2.356 (0.784, 7.078)		2.852 (2.136, 3.809)	2.160 (1.520, 3.069)	16.115 (7.309, 35.533)	16.922 (5.803, 49.352)	
Size of LMA			0.510					0.718
3	1.000 (Ref)	1.000 (Ref)		1.000 (Ref)	1.000 (Ref)	1.000 (Ref)	1.000 (Ref)	
4 or 5	0.961 (0.567, 1.628)	5.076 (0.000, Inf)		1.118 (0.659, 1.896)	0.841 (0.393, 1.801)	0.301 (0.086, 1.061)	0.243 (0.056, 1.056)	
Anesthetic method			0.581					0.787
Inhalation	1.000 (Ref)	1.000 (Ref)		1.000 (Ref)	1.000 (Ref)	1.000 (Ref)	1.000 (Ref)	
Intravenous	0.817 (0.525, 1.272)	0.485 (0.144, 1.639)		0.904 (0.597, 1.369)	0.805 (0.436, 1.486)	0.514 (0.182, 1.457)	0.456 (0.127, 1.641)	
Number of attempts			0.472					0.776
1	1.000 (Ref)	1.000 (Ref)		1.000 (Ref)	1.000 (Ref)	1.000 (Ref)	1.000 (Ref)	
2	1.687 (0.965, 2.951)	0.001 (0.000, Inf)		1.784 (1.044, 3.050)	1.577 (0.742, 3.352)	0.000 (0.000, 0.000)	0.000 (0.000, 0.000)	
3	3.286 (1.161, 9.296)			2.693 (0.920, 7.884)	0.461 (0.045, 4.757)	7.355 (1.456, 37.160)	3.384 (0.410, 27.908)	
Duration of anesthesia	1.072 (0.899, 1.278)	0.711 (0.382, 1.322)	0.672	1.035 (0.875, 1.224)	0.903 (0.734, 1.111)	1.111 (0.768, 1.608)	1.061 (0.709, 1.588)	0.726
<0.88	1.000 (Ref)	1.000 (Ref)	0.421	1.000 (Ref)	1.000 (Ref)	1.000 (Ref)	1.000 (Ref)	0.582
0.88 to 1.15	1.466 (0.975, 2.206)	0.888 (0.194, 4.059)		1.530 (1.032, 2.270)	1.365 (0.831, 2.242)	0.922 (0.380, 2.237)	1.099 (0.399, 3.026)	
1.15 to 1.45	1.325 (0.884, 1.984)	0.859 (0.172, 4.290)		1.364 (0.923, 2.015)	1.356 (0.828, 2.222)	0.719 (0.298, 1.735)	0.909 (0.323, 2.560)	
1.45 to 1.98	1.327 (0.888, 1.984)	1.469 (0.351, 6.145)		1.349 (0.911, 1.999)	1.522 (0.936, 2.476)	1.192 (0.535, 2.655)	1.503 (0.606, 3.728)	
≥1.98	1.153 (0.763, 1.743)	0.355 (0.082, 1.531)		1.080 (0.722, 1.614)	0.781 (0.476, 1.282)	1.041 (0.449, 2.412)	1.072 (0.418, 2.752)	
Supine cuff	1.018	1.022	0.556	1.017	1.018	1.042	1.048	0.757

pressure	(1.003, 1.033)	(0.971, 1.075)		(1.003, 1.031)	(0.999, 1.037)	(1.010, 1.075)	(1.011, 1.087)	
<20	1.000 (Ref)	1.000 (Ref)	0.662	1.000 (Ref)	1.000 (Ref)	1.000 (Ref)	1.000 (Ref)	0.894
20 to 24	1.124 (0.711, 1.778)	1.816 (0.324, 10.192)		1.203 (0.772, 1.877)	2.050 (1.120, 3.754)	0.546 (0.149, 1.997)	0.558 (0.143, 2.181)	
24 to 30	1.621 (1.062, 2.476)	1.020 (0.202, 5.161)		1.649 (1.088, 2.500)	1.926 (1.091, 3.400)	1.545 (0.575, 4.153)	1.089 (0.368, 3.224)	
30 to 37	1.871 (1.227, 2.852)	1.657 (0.331, 8.297)		1.789 (1.183, 2.706)	2.059 (1.176, 3.607)	2.870 (1.155, 7.128)	1.772 (0.657, 4.780)	
≥37	1.484 (0.956, 2.305)	2.265 (0.462, 11.111)		1.524 (0.990, 2.347)	2.251 (1.222, 4.148)	1.774 (0.656, 4.799)	2.051 (0.686, 6.130)	
Lateral cuff pressure	1.009 (0.990, 1.028)	1.015 (0.954, 1.081)	0.453	1.008 (0.990, 1.027)	1.008 (0.984, 1.032)	1.004 (0.962, 1.047)	0.993 (0.945, 1.043)	0.622
<20	1.000 (Ref)	1.000 (Ref)	0.729	1.000 (Ref)	1.000 (Ref)	1.000 (Ref)	1.000 (Ref)	0.813
20 to 25	1.131 (0.737, 1.738)	0.439 (0.098, 1.970)		1.094 (0.723, 1.654)	0.821 (0.485, 1.388)	1.391 (0.512, 3.783)	0.773 (0.258, 2.320)	
25 to 30	1.300 (0.817, 2.070)	0.650 (0.120, 3.517)		1.350 (0.867, 2.102)	1.211 (0.688, 2.131)	0.879 (0.284, 2.723)	0.628 (0.179, 2.205)	
30 to 36	1.018 (0.639, 1.623)	0.354 (0.057, 2.190)		1.018 (0.647, 1.601)	0.706 (0.393, 1.267)	0.684 (0.223, 2.097)	0.549 (0.164, 1.839)	
≥36	1.294 (0.751, 2.231)	0.905 (0.134, 6.116)		1.247 (0.739, 2.106)	1.136 (0.581, 2.222)	1.294 (0.387, 4.323)	0.791 (0.206, 3.032)	
Absolute change in CP	1.009 (0.990, 1.028)	1.015 (0.954, 1.081)	0.760	1.008 (0.990, 1.027)	1.008 (0.984, 1.032)	1.004 (0.962, 1.047)	0.993 (0.945, 1.043)	0.781
<-6	1.000 (Ref)	1.000 (Ref)	0.729	1.000 (Ref)	1.000 (Ref)	1.000 (Ref)	1.000 (Ref)	0.716
-6 to -2	0.865 (0.553, 1.353)	1.000 (0.227, 4.410)		0.893 (0.583, 1.366)	1.023 (0.592, 1.766)	0.626 (0.219, 1.785)	0.914 (0.274, 3.052)	
-2 to 1	0.992 (0.654, 1.505)	1.605 (0.396, 6.497)		0.958 (0.642, 1.430)	1.271 (0.758, 2.129)	1.562 (0.656, 3.719)	2.310 (0.791, 6.748)	
1 to 6	1.108 (0.712, 1.725)	1.406 (0.310, 6.378)		1.115 (0.729, 1.706)	1.080 (0.628, 1.856)	1.005 (0.378, 2.675)	1.200 (0.371, 3.884)	
≥6	1.217 (0.785, 1.886)	1.331 (0.295, 6.000)		1.225 (0.803, 1.867)	1.285 (0.742, 2.224)	0.940 (0.345, 2.562)	1.144 (0.333, 3.930)	
Relative change in CP	1.001 (0.996, 1.005)	0.998 (0.989, 1.008)	0.807	1.000 (0.996, 1.005)	0.999 (0.995, 1.004)	1.001 (0.990, 1.012)	0.997 (0.984, 1.010)	0.778
<-21.40	1.000 (Ref)	1.000 (Ref)	0.733	1.000 (Ref)	1.000 (Ref)	1.000 (Ref)	1.000 (Ref)	0.705
-21.40 to -7.14	0.851 (0.562, 1.289)	1.265 (0.330, 4.844)		0.850 (0.575, 1.256)	0.929 (0.567, 1.523)	0.976 (0.377, 2.523)	1.306 (0.426, 4.006)	
-7.14 to 4.55	0.835 (0.554,	0.891 (0.190,		0.777 (0.523,	0.876 (0.527,	1.544 (0.647,	2.262 (0.786,	

	1.257)	4.177)		1.154)	1.456)	3.682)	6.508)	
4.55 to 25.00	1.111 (0.738, 1.672)	1.255 (0.294, 5.357)		1.119 (0.756, 1.655)	0.975 (0.595, 1.598)	1.069 (0.418, 2.731)	1.254 (0.409, 3.846)	
≥25.00	1.171 (0.771, 1.781)	1.244 (0.265, 5.834)		1.127 (0.752, 1.691)	1.037 (0.610, 1.765)	1.186 (0.433, 3.244)	1.334 (0.388, 4.590)	
Cumulative absolute change in CP	1.010 (0.998, 1.023)	1.016 (0.978, 1.055)	0.619	1.010 (0.998, 1.022)	1.011 (0.996, 1.026)	1.018 (0.990, 1.046)	1.011 (0.979, 1.044)	0.710
<-7.83	1.000 (Ref)	1.000 (Ref)	0.811	1.000 (Ref)	1.000 (Ref)	1.000 (Ref)	1.000 (Ref)	0.818
-7.83 to 2.53	1.078 (0.712, 1.632)	1.825 (0.447, 7.449)		1.127 (0.763, 1.666)	1.113 (0.679, 1.825)	0.747 (0.295, 1.892)	0.558 (0.191, 1.633)	
-2.53 to 1.20	0.941 (0.622, 1.423)	1.135 (0.240, 5.372)		0.884 (0.592, 1.321)	0.990 (0.594, 1.653)	1.528 (0.693, 3.368)	1.708 (0.687, 4.247)	
1.20 to 6.77	1.109 (0.730, 1.684)	1.453 (0.335, 6.301)		1.132 (0.759, 1.687)	0.960 (0.579, 1.593)	0.687 (0.269, 1.755)	0.664 (0.232, 1.895)	
≥6.77	1.357 (0.884, 2.085)	1.469 (0.291, 7.417)		1.363 (0.906, 2.050)	1.250 (0.745, 2.098)	1.247 (0.510, 3.045)	0.957 (0.333, 2.749)	
Cumulative relative change in CP	1.002 (0.999, 1.005)	0.999 (0.994, 1.004)	0.549	1.002 (0.999, 1.004)	1.000 (0.998, 1.003)	1.005 (0.998, 1.012)	1.001 (0.994, 1.008)	0.638
<-28.40	1.000 (Ref)	1.000 (Ref)	0.762	1.000 (Ref)	1.000 (Ref)	1.000 (Ref)	1.000 (Ref)	0.797
-28.40 to -9.17	0.894 (0.596, 1.342)	1.242 (0.337, 4.579)		0.876 (0.594, 1.292)	0.902 (0.551, 1.476)	1.177 (0.472, 2.937)	1.189 (0.422, 3.348)	
-9.17 to 4.0	0.777 (0.517, 1.167)	1.218 (0.291, 5.096)		0.738 (0.497, 1.096)	0.810 (0.492, 1.335)	1.584 (0.682, 3.680)	1.843 (0.706, 4.812)	
4.0 to 28.8	1.120 (0.751, 1.671)	1.024 (0.244, 4.294)		1.092 (0.743, 1.607)	0.887 (0.545, 1.444)	1.104 (0.444, 2.744)	0.924 (0.319, 2.680)	
≥28.8	1.159 (0.763, 1.760)	1.777 (0.323, 9.778)		1.174 (0.788, 1.749)	1.152 (0.689, 1.925)	1.078 (0.401, 2.896)	1.267 (0.413, 3.890)	

Ambu-F, Ambu AuraFlex; CP, cuff pressure; FLMA, laryngeal mask airway Flexible; LMA, laryngeal mask airway; N.A., not available; POST, postoperative sore throat; Tuoren-ED, Tuoren Esophagus Drainage Type I; Tuoren-R, Tuoren Reinforced Type.

All logistic and multinomial models were adjusted for age, age squared, female sex, smoking, and BMI. Additionally, each model was further adjusted for type of LMA, size of LMA, anesthetic method, and number of attempts for duration of anesthesia and supine cuff pressure, and were adjusted for type of LMA, size of LMA, anesthetic method, number of attempts, and supine cuff pressure for lateral cuff pressure, position-induced absolute and relative change in CP, and cumulative absolute and relative change in CP. P for interaction was calculated by comparing the full logistic or multinomial models with and without the smoking-interaction term *via* likelihood ratio test. The quintiles of each variable were determined by stacking all completed datasets. All results were pooled based on Rubin's rules.

Table S12. Supine CP-stratified adjusted odds ratios for associations between segmented position-induced change and cumulative change in CP and acute incident POST based on 50 imputations

Factors	Segment	< 28 cmH_2O		≥ 28 cmH_2O		*P* for interaction
		OR (95% CI)	P value	OR (95% CI)	P value	
Absolute change in CP						0.112
	< -6.25	1.178 (0.983, 1.413)	0.076	1.033 (0.967, 1.103)	0.333	
	-6.25 to -0.52	0.892 (0.783, 1.016)	0.085	0.973 (0.881, 1.074)	0.583	
	-0.52 to 5.79	1.124 (1.022, 1.237)	0.017	1.049 (0.942, 1.168)	0.383	
	≥ 5.79	0.889 (0.810, 0.976)	0.014	1.051 (0.922, 1.198)	0.459	
Relative change in CP						0.244
	< -22.14	1.007 (0.981, 1.033)	0.621	1.019 (0.989, 1.049)	0.215	
	-22.14 to -1.48	0.984 (0.951, 1.017)	0.330	0.989 (0.962, 1.016)	0.408	
	-1.48 to 27.45	1.021 (1.000, 1.043)	0.049	1.019 (0.995, 1.045)	0.127	
	≥ 27.45	0.985 (0.971, 0.999)	0.033	0.993 (0.932, 1.058)	0.834	
Cumulative relative change in CP						0.057
	< -31.00	1.012 (0.998, 1.026)	0.085	1.009 (0.995, 1.024)	0.203	
	-31.00 to 0.41	0.984 (0.964, 1.003)	0.105	0.995 (0.979, 1.012)	0.584	
	0.41 to 53.11	1.007 (0.996, 1.019)	0.190	1.009 (0.994, 1.024)	0.222	
	≥ 53.11	0.998 (0.991, 1.005)	0.595	1.021 (0.978, 1.065)	0.339	

CP, cuff pressure; POST, postoperative sore throat.

All logistic models were adjusted for age, age squared, female sex, smoking, and BMI. Additionally, each model was further adjusted for type of LMA, size of LMA, anesthetic method, number of attempts, and supine cuff pressure for position-induced absolute and relative change in CP, and cumulative relative change in CP. Supine cuff pressure was categorized by its median value of 28 cmH_2O. P for interaction was calculated by comparing the full logistical models with and without the supine CP-interaction term *via* likelihood ratio tests. The median of supine cuff pressure and inflection points for segmentation were determined by stacking all completed datasets. All results were pooled based on Rubin’s rules.

Table S13. Supine CP-stratified adjusted odds ratios for associations between segmented position-induced change and cumulative change in CP and acute mild POST based on 50 imputations

Factors	Segment	< 28 cmH_2O		≥ 28 cmH_2O		*P* for interaction
		OR (95% CI)	P value	OR (95% CI)	P value	
Absolute change in CP						0.111
	< -6.44	1.214 (0.997, 1.478)	0.054	1.039 (0.969, 1.113)	0.284	
	-6.44 to -0.52	0.859 (0.753, 0.980)	0.023	0.963 (0.872, 1.065)	0.464	
	-0.52 to 5.79	1.155 (1.046, 1.275)	0.004	1.074 (0.960, 1.202)	0.210	
	≥ 5.79	0.889 (0.809, 0.978)	0.015	1.010 (0.874, 1.167)	0.891	
Relative change in CP						0.195
	< -23.79	1.009 (0.981, 1.037)	0.530	1.024 (0.991, 1.058)	0.155	
	-23.79 to -1.48	0.973 (0.942, 1.005)	0.097	0.987 (0.961, 1.013)	0.308	
	-1.48 to 27.45	1.030 (1.008, 1.053)	0.007	1.026 (1.000, 1.052)	0.054	
	≥ 27.45	0.982 (0.967, 0.997)	0.018	0.956 (0.881, 1.037)	0.277	
Cumulative relative change in CP						0.043
	< -34.05	1.013 (0.998, 1.028)	0.079	1.010 (0.994, 1.026)	0.213	
	-34.05 to -0.41	0.979 (0.961, 0.998)	0.032	0.994 (0.978, 1.011)	0.496	
	-0.41 to 50.05	1.013 (1.000, 1.025)	0.044	1.013 (0.997, 1.029)	0.107	
	≥ 50.05	0.996 (0.988, 1.004)	0.335	0.999 (0.948, 1.053)	0.966	

CP, cuff pressure; POST, postoperative sore throat.

For POST severity, threshold effect analyses were conducted for mild pain based on the binary logistic models owing to insignificant non-linear relationships between all position-induced change and cumulative change metrics in CP and moderate-to-severe pain. All models were adjusted for age, age squared, female sex, smoking, and BMI. Additionally, each model was further adjusted for type of LMA, size of LMA, anesthetic method, number of attempts, and supine cuff pressure for position-induced absolute and relative change in CP, and cumulative relative change in CP. Supine cuff pressure was categorized by its median value of 28 cmH_2O. P for interaction was calculated by comparing the full multinomial models with and without the supine CP-interaction term *via* likelihood ratio tests. The median of supine cuff pressure and inflection points for segmentation were determined by stacking all completed datasets. All results were pooled based on Rubin's rules.

Table S14. Duration of anesthesia-stratified adjusted odds ratios for associations between segmented position-induced change and cumulative change in CP and acute POST incidence based on 50 imputations

Factors	Segment	< 1.3 h		≥ 1.3 h		*P* for interaction
		OR (95% CI)	P value	OR (95% CI)	P value	
Absolute change in CP						0.037
	< -6.25	1.024 (0.942, 1.113)	0.583	1.081 (0.990, 1.181)	0.084	
	-6.25 to -0.52	0.981 (0.878, 1.096)	0.730	0.910 (0.815, 1.015)	0.091	
	-0.52 to 5.79	1.049 (0.948, 1.159)	0.354	1.146 (1.041, 1.261)	0.006	
	≥ 5.79	0.921 (0.827, 1.025)	0.131	0.951 (0.863, 1.047)	0.306	
Relative change in CP						0.031
	< -22.14	1.005 (0.977, 1.033)	0.749	1.014 (0.988, 1.041)	0.302	
	-22.14 to -1.48	0.994 (0.964, 1.024)	0.683	0.982 (0.954, 1.011)	0.219	
	-1.48 to 27.45	1.013 (0.992, 1.035)	0.229	1.028 (1.006, 1.050)	0.012	
	≥ 27.45	0.975 (0.955, 0.996)	0.018	0.992 (0.975, 1.010)	0.376	
Cumulative relative change in CP						0.055
	< -31.00	1.017 (0.978, 1.057)	0.402	1.010 (0.999, 1.021)	0.068	
	-31.00 to 0.41	0.991 (0.971, 1.011)	0.386	0.986 (0.969, 1.003)	0.115	
	0.41 to 53.11	1.006 (0.992, 1.020)	0.428	1.013 (1.002, 1.025)	0.019	
	≥ 53.11	0.955 (0.908, 1.004)	0.072	0.999 (0.993, 1.004)	0.673	

CP, cuff pressure; POST, postoperative sore throat.

All logistic models were adjusted for age, age squared, female sex, smoking, and BMI. Additionally, each model was further adjusted for type of LMA, size of LMA, anesthetic method, number of attempts, and supine cuff pressure for position-induced absolute and relative change in CP, and cumulative absolute and relative change in CP. Duration of anesthesia was categorized by its median value of 1.3 hours. P for interaction was calculated by comparing the full logistical models with and without the duration of anesthesia-interaction term *via* likelihood ratio tests. The median of anesthesia duration and inflection points for segmentation were determined by stacking all completed datasets. All results were pooled based on Rubin’s rules.

Table S15. Duration of anesthesia-stratified adjusted odds ratios for associations between segmented position-induced change and cumulative change in CP and acute mild POST based on 50 imputations

Factors	Segment	< 1.3 h		≥ 1.3 h		*P* for interaction
		OR (95% CI)	P value	OR (95% CI)	P value	
Absolute change in CP						0.082
	< -6.44	1.037 (0.949, 1.133)	0.422	1.085 (0.987, 1.192)	0.090	
	-6.44 to -0.52	0.957 (0.856, 1.069)	0.434	0.904 (0.809, 1.010)	0.074	
	-0.52 to 5.79	1.081 (0.975, 1.198)	0.137	1.158 (1.048, 1.280)	0.004	
	≥ 5.79	0.908 (0.813, 1.015)	0.089	0.931 (0.838, 1.034)	0.180	
Relative change in CP						0.097
	< -23.79	1.008 (0.978, 1.039)	0.588	1.015 (0.987, 1.044)	0.293	
	-23.79 to -1.48	0.989 (0.961, 1.018)	0.439	0.978 (0.951, 1.006)	0.127	
	-1.48 to 27.45	1.019 (0.996, 1.041)	0.100	1.033 (1.010, 1.056)	0.004	
	≥ 27.45	0.974 (0.954, 0.995)	0.015	0.985 (0.966, 1.005)	0.144	
Cumulative relative change in CP						0.057
	< -34.05	1.025 (0.978, 1.075)	0.294	1.010 (0.999, 1.022)	0.078	
	-34.05 to -0.41	0.989 (0.970, 1.008)	0.265	0.983 (0.967, 1.000)	0.052	
	-0.41 to 50.05	1.010 (0.995, 1.025)	0.197	1.017 (1.005, 1.030)	0.007	
	≥ 50.05	0.954 (0.908, 1.002)	0.059	0.997 (0.989, 1.004)	0.406	

CP, cuff pressure; POST, postoperative sore throat.

For POST severity, threshold effect analyses were conducted for mild pain based on the binary logistic models owing to insignificant non-linear relationships between all position-induced change and cumulative change metrics in CP and moderate-to-severe pain. All models were adjusted for age, age squared, female sex, smoking, and BMI. Additionally, each model was further adjusted for type of LMA, size of LMA, anesthetic method, number of attempts, and supine cuff pressure for position-induced absolute and relative change in CP, and cumulative relative change in CP. Duration of anesthesia was categorized by its median value of 1.3 hours. P for interaction was calculated by comparing the full multinomial models with and without the anesthesia duration-interaction term *via* likelihood ratio tests. The median of anesthesia duration and inflection points for segmentation were determined by stacking all completed datasets. All results were pooled based on Rubin's rules.

Table S16. Baseline clinical characteristics of patients in the sensitivity analysis using the "Unknown"-category method

Variables, N. (%)	Overall (N = 1,363)	Non-POST (N = 823)	POST (N =540)	*P* value	POST severity: Mild (N = 470)	POST severity: Moderate-to-severe (N = 70)	*P* value [a]
Age, years	44.46 (13.34)	44.01 (13.52)	45.14 (13.03)	0.126	0.51 (13.06)	1.81 (12.88)	0.232
Female	776 (56.9)	427 (51.9)	349 (64.6)	<0.001	296 (63.0)	53 (75.7)	<0.001
Smoking				<0.001			<0.001
No	583 (42.8)	388 (47.1)	195 (36.1)		183 (38.9)	12 (17.1)	
Yes	77 (5.6)	58 (7.0)	19 (3.5)		18 (3.8)	1 (1.4)	
Unknown	703 (51.6)	377 (45.8)	326 (60.4)		269 (57.2)	57 (81.4)	
Weight, kg	62.94 (11.64)	63.17 (11.45)	62.58 (11.93)	0.358	62.86 (12.22)	60.71 (9.62)	0.233
Height, cm	164.74 (7.94)	165.37 (7.91)	163.78 (7.88)	<0.001	163.94 (8.00)	162.71 (6.98)	0.001
BMI, kg/m^2	23.09 (3.25)	23.00 (3.20)	23.21 (3.32)	0.243	23.26 (3.37)	22.88 (2.97)	0.336
Type of LMA				<0.001			<0.001[b]
FLMA	602 (44.2)	432 (52.5)	170 (31.5)		162 (34.5)	8 (11.4)	
Ambu-F	35 (2.6)	28 (3.4)	7 (1.3)		7 (1.5)	0 (0.0)	
Tuoren-R	198 (14.5)	119 (14.5)	79 (14.6)		70 (14.9)	9 (12.9)	
Tuoren-ED	528 (38.7)	244 (29.6)	284 (52.6)		231 (49.1)	53 (75.7)	
Size of LMA				<0.001			<0.001
3	750 (55.0)	415 (50.4)	335 (62.0)		281 (59.8)	54 (77.1)	
4 or 5	613 (45.0)	408 (49.6)	205 (38.0)		189 (40.2)	16 (22.9)	
Anesthetic method				<0.001			<0.001
Inhalation	117 (8.6)	72 (8.7)	45 (8.3)		40 (8.5)	5 (7.1)	
Intravenous	628 (46.1)	419 (50.9)	209 (38.7)		201 (42.8)	8 (11.4)	
Unknown	618 (45.3)	332 (40.3)	286 (53.0)		229 (48.7)	57 (81.4)	
Surgery ear				<0.001			<0.001[c]
Right	439 (32.2)	276 (33.5)	163 (30.2)		151 (32.1)	12 (17.1)	
Left	441 (32.4)	294 (35.7)	147 (27.2)		136 (28.9)	11 (15.7)	
Both	15 (1.1)	10 (1.2)	5 (0.9)		5 (1.1)	0 (0.0)	
Unknown	468 (34.3)	243 (29.5)	225 (41.7)		178 (37.9)	47 (67.1)	

Number of attempts				0.115			0.012 [c]
1	1280 (93.9)	780 (94.8)	500 (92.6)		433 (92.1)	67 (95.7)	
2	65 (4.8)	36 (4.4)	29 (5.4)		29 (6.2)	0 (0.0)	
≥3	18 (1.3)	7 (0.9)	11 (2.0)		8 (1.7)	3 (4.3)	
Duration of anesthesia, min	1.28 [0.97,1.78]	1.28 [0.95,1.82]	1.30 [0.97,1.72]	0.887	1.28 [0.97,1.72]	1.38 [0.95,1.79]	0.954
Intracuff pressure, cmH_2O							
Supine	27.95 (8.74)	27.38 (8.79)	28.83 (8.59)	0.003	28.58 (8.51)	30.50 (8.99)	0.003
Lateral	27.49 (9.18)	26.71 (9.10)	28.67 (9.18)	<0.001	28.43 (9.17)	30.29 (9.15)	<0.001
Absolute Δ	0.00 [-4.00,4.00]	0.00 [-4.00,4.00]	0.00 [-4.00,4.00]	0.169	0.00 [-4.00,4.00]	0.00 [-2.00,4.00]	0.382
Relative Δ	0.00 [-16.67,18.18]	0.00 [-17.80,18.02]	0.00 [-14.46,18.18]	0.224	0.00 [-15.38,20.00]	0.00 [-9.84,10.83]	0.477
Cumulative absolute Δ	0.00 [-6.30,5.13]	0.00 [-6.42,4.87]	0.00 [-5.95,5.60]	0.123	0.00 [-6.10,5.60]	0.00 [-3.92,5.32]	0.304
Cumulative relative Δ	0.00 [-20.94,21.74]	0.00 [-22.72,20.54]	0.00 [-19.47,23.59]	0.181	0.00 [-20.50,23.98]	0.00 [-13.88,18.91]	0.406

Ambu-F, Ambu AuraFlex; BMI, body mass index; FLMA, laryngeal mask airway Flexible; LMA, laryngeal mask airway; POST, postoperative sore throat; Tuoren-ED, Tuoren Esophagus Drainage Type I; Tuoren-R, Tuoren Reinforced Type.

[a] P value was calculated by comparing difference between three POST severity groups, including no pain, mild pain, and moderate-to-severe pain.

[b] Pearson’s chi-squared test using continuity correction.

[c] Fisher's exact test, in which P values were calculated using Monte Carlo simulation with 100,000 replicates.

Absolute and relative Δ refer to absolute and relative change in CP, whereas cumulative absolute and relative Δ refer to cumulative absolute and relative change in CP, respectively.

Table S17. Adjusted odds ratios for associations between intraoperative factors and POST incidence and severity in the sensitivity analysis using the "Unknow"-category method

Factors	POST incidence		POST severity			
			Mild		Moderate-to-severe	
	OR (95% CI)	*P* value	OR (95% CI)	*P* value	OR (95% CI)	*P* value
Type of LMA						
FLMA	1.000 (Ref)		1.000 (Ref)		1.000 (Ref)	
Ambu-F	0.569 (0.222, 1.278)	0.199	0.585 (0.247, 1.387)	0.224	N.A.	N.A.
Tuoren-R	1.784 (1.260, 2.520)	0.001	1.631 (1.143, 2.328)	0.007	5.359 (1.968, 14.594)	0.001
Tuoren-ED	3.090 (2.328, 4.118)	<0.001	2.640 (1.967, 3.543)	<0.001	12.471 (5.581, 27.866)	<0.001
Size of LMA						
3	1.000 (Ref)		1.000 (Ref)		1.000 (Ref)	
4 or 5	1.030 (0.619, 1.715)	0.910	1.174 (0.696, 1.980)	0.549	0.320 (0.086, 1.192)	0.089
Anesthetic method						
Inhalation	1.000 (Ref)		1.000 (Ref)		1.000 (Ref)	
Intravenous	0.786 (0.520, 1.196)	0.255	0.844 (0.550, 1.295)	0.438	0.305 (0.098, 0.947)	0.040
Unknown	0.760 (0.420, 1.381)	0.365	0.653 (0.357, 1.194)	0.166	12.117 (7.953, 18.459)	<0.001
Number of attempts						
1	1.000 (Ref)		1.000 (Ref)		1.000 (Ref)	
2	1.525 (0.894, 2.586)	0.118	1.677 (0.992, 2.834)	0.054	N.A.	N.A.
3	3.363 (1.222, 9.824)	0.021	2.864 (0.974, 8.419)	0.056	9.024 (1.705, 47.765)	0.010
Duration of anesthesia	1.012 (0.865, 1.183)	0.881	1.010 (0.860, 1.187)	0.901	1.032 (0.721, 1.477)	0.863
Intracuff pressure						
Supine	1.019 (1.006, 1.033)	0.006	1.016 (1.003, 1.030)	0.020	1.038 (1.006, 1.070)	0.018
Lateral	1.012 (0.995, 1.030)	0.178	1.012 (0.994, 1.031)	0.180	1.005 (0.964, 1.048)	0.803
Absolute change	1.012 (0.995, 1.030)	0.178	1.012 (0.994, 1.031)	0.180	1.005 (0.964, 1.048)	0.803
Relative change	1.001 (0.997, 1.004)	0.736	1.000 (0.997, 1.004)	0.784	1.001 (0.991, 1.011)	0.842
Cumulative absolute change	1.013 (1.002, 1.024)	0.024	1.012 (1.001, 1.024)	0.038	1.017 (0.989, 1.045)	0.232
Cumulative relative change	1.001 (0.999, 1.003)	0.212	1.001 (0.999, 1.003)	0.325	1.003 (0.999, 1.008)	0.154

Ambu-F, Ambu AuraFlex; FLMA, LMA Flexible; LMA, laryngeal mask airway; N.A., not available; POST, postoperative sore throat; OW, overlap weighting; Tuoren-ED, Tuoren Esophagus Drainage Type I; Tuoren-R, Tuoren Reinforced Type.

All models were adjusted for age, age squared, female sex, smoking, and BMI. Additionally, each model was further adjusted for type of LMA, size of LMA, anesthetic method, and number of attempts for duration of anesthesia and supine cuff pressure, and adjusted for type of LMA, size of LMA, anesthetic method, number of attempts, and supine cuff pressure for lateral cuff pressure, position-induced absolute and relative change in CP, and cumulative absolute and relative change in CP.
